# AI-2 Induces Urease Expression Through Downregulation of Orphan Response Regulator HP1021 in *Helicobacter pylori*

**DOI:** 10.3389/fmed.2022.790994

**Published:** 2022-04-01

**Authors:** Huang Yang, Xiaoxing Huang, Xiaochuan Zhang, Xiaoyan Zhang, Xiaohong Xu, Feifei She, Yancheng Wen

**Affiliations:** ^1^Key Laboratory of Gastrointestinal Cancer (Fujian Medical University), Ministry of Education, Fuzhou, China; ^2^Fujian Key Laboratory of Tumor Microbiology, Department of Medical Microbiology, Fujian Medical University, Fuzhou, China; ^3^Fujian Medical University Union Hospital, Fuzhou, China

**Keywords:** *Helicobacter pylori*, quorum sensing, LuxS, AI-2, urease, HP1021

## Abstract

*Helicobacter pylori* causes gastric infections in more than half of the world's population. The bacterium's survival in the stomach is mediated by the abundant production of urease to enable acid acclimation. In this study, our transcriptomic analysis demonstrated that the expression of urease structural proteins, UreA and UreB, is induced by the autoinducer AI-2 in *H. pylori*. We also found that the orphan response regulator HP1021 is downregulated by AI-2, resulting in the induction of urease expression. HP1021 represses the expression of urease by directly binding to the promoter region of *ureAB*, ranging from −47 to +3 with respect to the transcriptional start site. The study findings suggest that quorum sensing via AI-2 enhances acid acclimation when bacterial density increases, and might enable bacterial dispersal to other sites when entering gastric acid.

## Introduction

*Helicobacter pylori* is a microaerobic, gram-negative, gastric pathogen that colonizes more than half of the world's population (1, 2). Infection with *H. pylori* is closely related to the onset of many gastrointestinal diseases, including chronic atrophic gastritis, peptic ulcer, gastric cancer, and gastric mucosa-associated lymphoma (3). Many virulence factors are involved in the pathogenesis of *H*. *pylori*. For example, flagella enable the bacteria to penetrate the gastric mucosa and migrate to gastric epithelial cells (4, 5). Additionally, several outer membrane proteins, such as BabA, SabA, AlpA, AlpB, HopZ, HopQ, and OipA, are involved in bacterium-host interactions that facilitate bacterial adhesion to gastric epithelial cells (6–11). Further, *H*. *pylori* synthesizes and delivers cytotoxin-associated gene A (CagA) protein to gastric epithelial cells through a type IV secretion system (T4SS), which activates the signal transduction pathways involved in the development of gastric cancer (12). T4SS is critical to activating the proinflammatory response via delivery of ADP-heptose, an intermediate metabolite of lipopolysaccharide, to the host cell (13). Finally, *H*. *pylori* secretes another major toxin, vacuolating cytotoxin A (VacA), which enhances colonization by inducing apoptosis, autophagy, and cell death, as well as disruption of the cytoskeletal architecture in human gastric adenocarcinoma (AGS) cells (14–16).

Survival of *H. pylori* under harsh acidic conditions in the stomach is critical, *H*. *pylori* has developed a unique acid acclimation mechanism through the production of urease and regulation of its activity. Urease is a 550 kDa, multimeric protein that can hydrolyze urea to ammonia and carbonic acid (17). The urease gene cluster comprises two operons: *ureAB* and *ureIEFGH*. The *ureAB* operon encodes the two structural subunits of urease: UreA (26.5 kDa) and UreB (60.3 kDa). Encoded by the *ureIEFGH* operon, UreE, UreF, UreG, and UreH are four accessory proteins involved in facilitating nickel transfer and insertion into apo-urease (18), while UreI is an inner membrane protein that forms a proton-gated urea channel to increase the influx of urea in acidic conditions (19). After urea enters the cytoplasm, it is hydrolyzed by urease to generate ammonia, maintaining cytoplasmic pH homeostasis (20, 21).

Urease is abundant in *H. pylori*, accounting for ~8% of total protein, and is critical for the survival and growth of *H. pylori* in acidic gastric environments (22). Indeed, urease-deficient *H*. *pylori* are unable to achieve successful colonization in gastric epithelial cells (23–25). Acidic pH induces the expression of urease through the two-component system, ArsRS, which directly activates the expression of urease (26). A *cis*-encoded antisense sRNA, 5' *ureB*-sRNA downregulates *ureAB* transcription by enhancing transcriptional termination of UreAB mRNA (27, 28). Besides, NikR directly activates the transcription of *ureAB* by binding to the promoter region of *ureAB* in the presence of nickel (29). However, the regulation of urease expression by other factors remains to be elucidated.

Quorum sensing is a cell-to-cell communication mechanism utilized by bacteria to coordinate group behaviors, including bioluminescence production, biofilm formation, bacterial competence, toxin production, and motility (30–35). Quorum sensing involves the secretion and accumulation of extracellular chemical signals (named autoinducers) as bacterial density increases, recognition of the autoinducers, signal transduction, and regulation of bacterial behavior. Gram-negative bacteria secrete and recognize autoinducers such as *N*-acylated homoserine lactone (AHL), which is the most common class of autoinducer (36, 37). Another autoinducer present in most gram-negative bacteria, AI-2, is derived from 4,5-dihydroxy-2,3-pentanedione (DPD), which is synthesized by LuxS and has been proposed to be involved in interspecies communication. LuxS is reportedly responsible for the synthesis of AI-2 in *H. pylori* (38–40). Studies have shown that AI-2 serves as a chemical repellent and also participates in the regulation of *H. pylori* chemotaxis and motility (41–43). Further, AI-2 inhibits biofilm formation and promotes bacterial dispersal in *H. pylori* (44, 45). We recently reported that AI-2 inhibits CagA expression and bacterial adhesion, thus attenuating the *H. pylori*-induced inflammatory response in gastric epithelial cells (46). Taken together, these results suggest that AI-2 plays an important role in the regulation of *H*. *pylori* pathogenesis.

Therefore, the goal of the current study was to investigate the role of AI-2 in *H*. *pylori* gene regulation, thus determining the underlying mechanism that regulates urease expression and further elucidating the factors responsible for *H*. *pylori* virulence.

## Materials and Methods

### Bacterial Strains and Growth Conditions

*H. pylori* strain 26695 and its isogenic mutants Δ*hp1021*, Δ*fur*, Δ*hp0564*, Δ*hrcA*, Δ*rpoN*, and Δ*luxS*Δ*hp1021* were routinely maintained on Columbia blood agar plates supplemented with 5% sheep blood at 37 °C under microaerophilic conditions (5% O_2_, 10% CO_2_, and 85% N_2_). For liquid culture, *H*. *pylori* strains were cultured in Brucella broth (BD Biosciences, San Jose, CA, USA) supplemented with 10% fetal bovine serum (PAN Seratech, Aidenbach, Germany) (BB+FBS) with gentle agitation (120 rpm). Kanamycin (5 μg/mL) or chloramphenicol (4 μg/mL) were added to *H*. *pylori* isogenic or complemented mutant strains when necessary. The *Escherichia coli* strain BL21 (DE3) (Novagen, Darmstadt, Germany) was cultured in Luria broth (LB) under aerobic conditions at 37°C with agitation (230 rpm).

### Construction of *H. pylori* Strain 26695 Isogenic Mutants Δ*hp1021*, Δ*fur*, Δ*hp0564*, Δ*hrcA*, Δ*rpoN*, and Δ*luxS*Δ*hp1021*

Construction of *H*. *pylori* strain 26695 isogenic mutants was performed as previously described (46). Briefly, to construct the Δ*hp1021* mutant, primers *hp1021*-up-F and *hp1021*-up-R were used to amplify the upstream sequence of *hp1021* using the genomic DNA of *H*. *pylori* strain 26695 as the template; primers *hp1021*-down-F and *hp1021*-down-R were used to amplify the downstream sequence of *hp1021*; and primers *hp1021*-*aphA3*-F and *hp1021*-*aphA3*-R were used to amplify the kanamycin resistance gene cassette (*aphA*-3). AphA-3 was amplified from pHel3 purchased from Addgene (Addgene plasmid 102961) (47). Then, pBluescript II SK (–) (Stratagene, La Jolla, CA, USA) was linearized by digestion with KpnI and HindIII (New England Biolabs, Ipswich, MA, USA). The upstream and downstream fragments and the kanamycin resistance gene cassette were ligated to lineariz pBluescript II SK(-) using the ClonExpress Ultra One Step Cloning Kit (Vazyme Biotech, Nanjing, China), generating pBluescript-*hp1021*KO, which was subsequently transfected into *E. coli* DH5α. Colonies were selected on LB agar plates containing kanamycin (25 μg/mL). After confirmation by colony PCR and sequencing, pBluescript-*hp1021*KO was extracted and transformed to *H. pylori* strain 26695 by electroporation. The *hp1021* knockout mutant (Δ*hp1021*) was selected on Columbia agar plates supplemented with kanamycin (5 μg/mL), and deletion of *hp1021* was confirmed by DNA sequencing.

Construction of Δ*fur*, Δ*hp0564*, Δ*hrcA*, and Δ*rpoN* were performed as described above, with the following changes: primers *fur*-up-F, *fur*-up-R, *fur*-down-F, *fur*-down-R, *fur*-*aphA3*-F, and *fur*-*aphA3-*R were used to construct Δ*fur*; primers *hp0564*-up-F, *hp0564*-up-R, *hp0564*-down-F, *hp0564*-down-R, *hp0564*-*aphA3*-F, and *hp0564*-*aphA3*-R were used to construct Δ*hp0564*; primers *hrcA*-up-F, *hrcA*-up-R, *hrcA*-down-F, *hrcA*-down-R, *hrcA*-*aphA3*-F, and *hrcA*-*aphA3*-R were used to construct Δ*hrcA*; and primers *rpoN*-up-F, *rpoN*-up-R, *rpoN*-down-F, *rpoN*-down-R, *rpoN*-*aphA3*-F, and *rpoN*-*aphA3*-R were used to construct Δ*rpoN*.

Construction of Δ*luxS*Δ*hp1021* was performed by introducing upstream and downstream fragments of the *hp1021* coding region flanked by a non-polar chloramphenicol acetyltransferase gene (CAT) amplified from pHel2 into Δ*luxS*. The plasmid pHel2 purchased from Addgene (Addgene plasmid 102960). Primers *hp1021*-up-F1 and *hp1021*-up-R1, and *hp1021*-down-F1 and *hp1021*-down-R1 were used to amplify the *hp1021* upstream and downstream sequences, respectively. Primers *hp1021*-CAT-F and *hp1021*-CAT-R were used to amplify CAT. Allelic exchange of *hp1021* was performed as described above, and Columbia agar plates with chloramphenicol (4 μg/mL) were used to select Δ*luxS*Δ*hp1021* mutant colonies.

### Chromosomal Complementation of *Hp1021* in Δ*hp1021* (Δ*hp1021*/*Hp*1021^C^)

For complementation of HP1021 in Δ*hp1021*, the *hp1021* coding sequence ligated downstream of the *ureAB* promoter region was introduced to the chromosome in the intergenic region between *hp0204* and *hp0203*, as previously reported (48, 49). Primers *hp*1021^C^-up-F, and *hp*1021^C^-up-R, and *hp*1021^C^-down-F, and *hp*1021^C^-down-R, were used to amplify the sequences upstream and downstream of the insertion locus, respectively. Primers *hp*1021^C^-P*ureA*-F and *hp*1021^C^-P*ureA*-R were used to amplify the *ureA* promoter; primers *hp*1021^C^-F and *hp*1021^C^-R were used to amplify the *hp1021* coding sequence; and primers *hp*1021^C^-CAT-F and *hp*1021^C^-CAT-R were used to amplify CAT. Allelic exchange was performed as described above. Insertion of *hp1021*-CAT was selected with chloramphenicol (4 μg/mL) and confirmed by DNA sequencing. HP1021 expression was confirmed by evaluation of the HP1021 mRNA level.

### RNA Isolation

*H. pylori* strains were cultured on Columbia agar plates for 3 days, followed by collection of cells, resuspension in Brucella broth with an initial OD_600_ of ~0.2, and culturing for another 8 h until the cells reached the exponential growth phase. To prepare the Δ*luxS*+AI-2 mutant, *H*. *pylori* Δ*luxS* was resuspended in Brucella broth and cultured for 4 h, then 80 μM AI-2 was added, followed by culturing for another 4 h. Bacterial cells were subsequently collected for total RNA isolation using TRIzol reagent (Invitrogen, Carlsbad, CA, USA), according to the manufacturer's instructions. RNA quality was confirmed by gel electrophoresis, and the RNA concentration was quantified using a NanoDrop One spectrophotometer (Thermo Fisher Scientific, Waltham, MA, USA).

### Reverse Transcription and Quantitative PCR (QPCR) Analysis

cDNA synthesis was performed using the HiScript Q RT SuperMix for qPCR (Vazyme Biotech) according to the manufacturer's instructions. Briefly, 1 μg isolated total RNA was treated with 4 × gDNA Wiper Mix to eliminate genomic DNA contamination. Reverse transcription was performed by adding 5 × HiScript qRT SuperMix II. To quantify the UreA, UreB, and HP1021 mRNA levels, qPCR was performed using the ChamQ Universal SYBR qPCR Master Mix (Vazyme Biotech) with the Mx3000P QPCR system (Agilent Technologies, Santa Clara, CA) according to the manufacturer's instructions. PCR amplification consisted of an initial denaturation step for 60 s at 95°C, followed by a 40-cycle reaction: 95°C for 5 s, 60°C for 34 s. Gene expression levels were calculated using the 2^−ΔΔCT^ method, while 16S rRNA was used as an endogenous control gene. qPCR was conducted in accordance with the guidelines (50). Relative RNA levels were presented as gene expression levels of the target genes normalized to that of the wild type. The primers used for each target gene are listed in Supplementary Table 1.

### Transcriptomic Analysis

Overnight cultures of *H*. *pylori* wild type or Δ*luxS* mutant were resuspended in Brucella broth with an initial OD_600_ of ~0.2 and cultured for 8 h until the log phase of growth. RNA samples were prepared using the RNeasy Mini Kit (Qiagen, Hilden, Germany) according to the manufacturer's instructions. Four samples containing two biological replicates were included in the transcriptomic analysis. RNA quality was verified using the 2100 Bioanalyzer (Agilent Technologies). All RNA sequencing (RNAseq) and alignment procedures were performed by Novogene Bioinformatics Technology Co., Ltd. (Beijing, China). The reads were mapped against the reference genome of *H*. *pylori* strain 26695 (51). The relative RNA level was measured in reads per kilobase per million mapped reads (RPKM). Genes were considered LuxS-regulated genes when |log_2_fold change| > 1.0, while the *P-value* < 0.05.

### SDS-PAGE and Western Blotting

For SDS-PAGE, *H. pylori* strains were cultured as described above, cells were collected, and bacterial lysates were prepared by ultrasonication. Cellular debris was removed by centrifugation, and the protein concentration was quantified using the BCA Protein Assay Kit (Beyotime Biotech, Shanghai, China). After boiling, 10 μg cell lysate for each sample plus a 180-kDa pre-stained protein marker (Vazyme) was resolved on a 10% SDS-PAGE gel and stained with Coomassie Blue Super Fast Staining Solution (Beyotime Biotech). Gels were analyzed using a GS-900 calibrated densitometer (Bio-Rad Laboratories, Hercules, CA, USA) to determine UreA protein levels.

For western blotting, proteins were transferred to a PVDF membrane (Millipore, Billerica, MA, USA) and blocked for 1 h with TBS-T buffer (50 mM Tris, 150 mM NaCl, 0.05% Tween-20) containing 5% BSA (Medix, Shanghai, China). Anti-UreB primary antibody (1:50,000; Abcam, Shanghai, China) was added and incubated overnight at 4°C. After washing with TBS-T buffer three times, the membranes were incubated with anti-rabbit IgG, HRP-linked secondary antibody (1:2,000; Cell Signaling, Danvers, MA, USA) for 1 h at room temperature. The membranes were washed three times with TBS-T, and UreB protein signals were detected using a WesternBright ECL Kit (Advansta Inc., San Jose, CA, USA) and visualized using the ImageQuant LAS 4000 mini system (GE Life Sciences, Piscataway, NJ, USA).

### Urease Activity Assay

Urease activity of bacterial lysates were determined through measurement of ammonia production using phenol-hypochlorite methods. Briefly, bacteria were cultured in BB+FBS until logarithmic growth phase as described above, and were subsequently collected and resuspended in 300 μl HEPES buffer (pH 7.5). Cell lysates were obtained by sonication while the concentration was quantified using BCA Protein Assay Kit (Beyotime Biotech). For the determination of urease activity, 20 μl cell lysate was first incubated with the urea solution for 1 h at 37°C using urease activity detection kit (Solarbio, Beijing, China) according to the manufacturer's instructions. The phenol-hypochlorite reaction was then performed for 20 min at room temperature to detect the released ammonia. The absorbance at 630 nm was measured using an EnSight^TM^ Multimode Plate Reader (PerkinElmer, Waltham, MA, USA). The urease activity (U/mg protein) are expressed as μg NH_3_/min/mg protein.

### Expression and Purification of Strep-HP1021

For the expression of Strep-HP1021, the DNA fragment containing the coding region of *hp1021* was amplified with primers *hp*1021^CDS^-F and *hp*1021^CDS^-R, and cloned into pASK-IBA7plus (IBA Lifesciences, Göttingen, Germany). The resulting expression vector, pASK-IBA7-HP1021, was subsequently transfected into the *E. coli* strain BL21 (DE3). Colonies were selected on LB agar plates containing ampicillin, and the presence of the expression vector was confirmed by colony PCR and DNA sequencing. For induction and purification of Strep-HP1021, overnight cultures of *E. coli* strain BL21 (DE3) carrying the expression vector were washed and resuspended in 250 mL fresh LB medium. Then, the bacteria were cultured at 37°C with agitation (250 rpm) until the exponential growth phase with an OD_600_ of ~0.6. Tetracycline (0.2 μg/mL) was added to induce protein expression. After 3 h, cells were harvested by centrifugation at 6,000 rpm for 10 min at 4 °C. Bacterial cell lysates were obtained by ultrasonication, and Strep-HP1021 was purified using Strep-Tactin Sepharose resin (IBA Lifesciences) according to the manufacturer's instructions. The purified Strep-HP1021 was dialyzed using Tube-O-DIALYZER (G-Biosciences, St. Louis, MO, USA) with washing buffer (100 mM Tris-Cl, 150 mM NaCl, 10 mM EDTA). The purity of Strep-HP1021 was assessed by SDS-PAGE followed by Coomassie blue staining, and the Strep-HP1021 concentration was measured using the BCA Protein Assay Kit (Beyotime Biotech).

### Electrophoretic Mobility Shift Assay (EMSA)

The promoter region of *ureAB* was determined according to the transcriptional start site reported by Sharma et al. (52). A 252-bp DNA fragment containing the promoter region of *ureA* (ranging from −252 to −1 with respect to the start codon) was amplified by P*ureA*-F and P*ureA*-AF700-R, which was labeled at the 5'-end with Alexa Fluor 700 (AF700) (Thermo Fisher Scientific). EMSA was performed by incubating 12 fmol AF700-labeled DNA probe with increasing concentrations of Strep-HP1021 (0, 25, 50, 100, 200, 300 nM) in 20 μL reaction mixture (50 mM Tris-Cl, 250 mM KCl, and 5 mM DTT, pH 7.0) at 37°C for 30 min. For each sample, 100 ng/μL sheared salmon sperm DNA (Solarbio) were supplied. DNA probe without labeling was amplified with P*ureA*-F and P*ureA*-R and was used in excess (25-, 50-, and 100-fold concentrations) as a cold probe. The binding reaction was terminated by adding loading buffer (0.25% bromophenol blue, 0.25% xylene cyanol, and 40% dextrose) and the sample mixtures were resolved on a 4% native polyacrylamide gel. DNA bands were visualized using the Odyssey CLx imaging system (LI-COR Biosciences, Lincoln, NE, USA) with an excitation wavelength of 700 nm, and the binding between HP1021 and the AF700-labeled *ureA* promoter region was analyzed using Image Studio version 5.2 software (LI-COR Biosciences).

### DNase I Footprinting Assay

To identify the specific binding site for HP1021, a DNase I footprinting assay was performed. The promoter region of *ureA* was first amplified using P*ureA*-F and P*ureA*-R, and the obtained PCR product was then ligated to the pLB-Simple Vector using the Lethal Based Simple Fast Cloning Kit (Tiangen Biotech, Beijing, China) to construct pLB-P*ureA*. The promoter region of *ureA* was amplified using primers pLB-P*ureA*-F and pLB-P*ureA*-FAM-R, which was labeled at its 5'-end with 6-carboxyfluorescein (FAM) (Invitrogen). The FAM-labeled PCR product was then purified, and its concentration was quantified using a NanoDrop One spectrophotometer (Thermo Fisher Scientific). The DNase I footprinting assay was performed as previously described (53). Briefly, 50 ng probe was incubated with or without Strep-HP1021 (5 μg) in a total volume of 40 μL for 30 min at 37°C. For each sample, 100 ng/μL sheared salmon sperm DNA were supplied. Then, 10 μL solution containing 0.015 units DNase I (Promega, Madison, WI, USA) and 100 nmol CaCl_2_ was added, followed by incubation at 37°C for 1 min. The digestion was terminated by the addition of 140 μL stop solution (200 mM sodium acetate, 30 mM EDTA, and 0.15% SDS). Digested DNA was extracted with phenol/chloroform, precipitated with 70% ethanol, and finally dissolved in 10 μL HiDi formamide (Applied Biosystems, Foster City, CA, USA) with 1 μL GeneScan-LIZ 500 standard (Applied Biosystems). The samples were then analyzed by capillary electrophoresis using a 3730 DNA Analyzer (Applied Biosystems), while the HP1021 binding site was analyzed using Peak Scanner version 1.0 software (Applied Biosystems).

### Site-Directed Mutagenesis

The HP1021 binding site on *ureA* promoter were mutated using Mut Express MultiS Fast Mutagenesis Kit V2 (Vazyme Biotech) according to the manufacturer's instructions. The plasmid pLB-P*ureA* containing the *ureA* promoter region was used as template while primers pLB-P*ureA*mt-F and pLB-P*ureA*mt-R (listed in Supplementary Table 1) carrying the substituted nucleotides were used. The resulting plasmid pLB-P*ureA*mt were confirmed by DNA sequencing. AF700-labled P*ureA*mt was obtained by PCR amplification using pLB-P*ureA*mt as template while P*ureA*-F and P*ureA*-AF700-R as primers.

### Statistical Analysis

All results were analyzed using GraphPad Prism version 7.0 software (GraphPad Software, La Jolla, CA, USA). Student's *t*-test was used to evaluate the statistical significance of differences between transcript levels of target genes in two groups. A *p*-value < 0.05 was considered statistically significant.

## Results

### Identification of LuxS-Regulated Genes Through Transcriptomic Profiling

**Figure 1:**
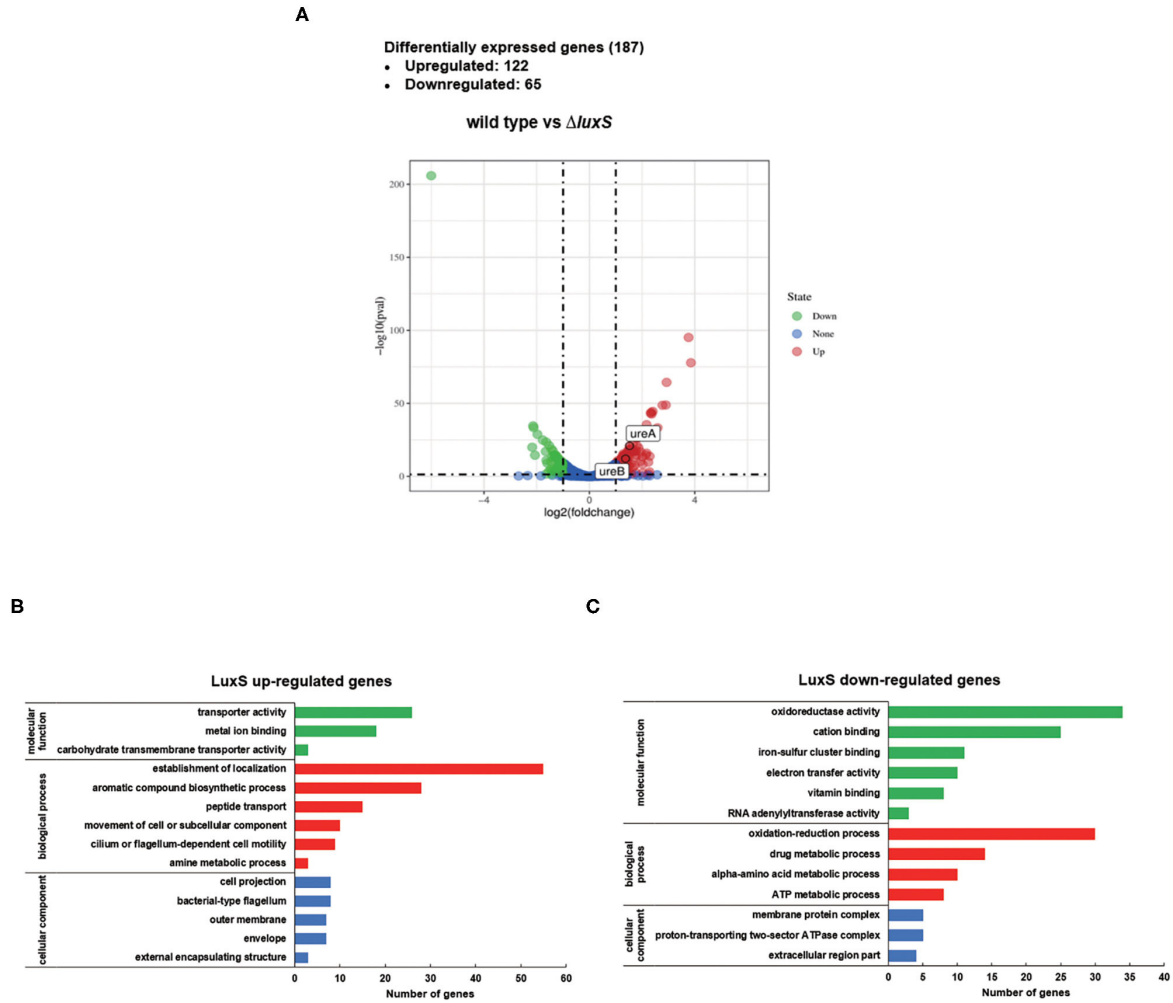
Differentially expressed genes (DEGs) regulated by LuxS identified by RNA sequencing. **(A)** Volcano plot comparing gene transcripts from wild type and Δ*luxS* mutant strains of *H. pylori*. The dashed transverse line indicates where *P-value* = 0.05 and the two vertical dotted lines represent |log_2_FoldChange| = 1. Genes of |log_2_FoldChange| > 1 and *P-value* < 0.05 are highlighted in red (significantly upregulated) and green (significantly downregulated). *ureA* and *ureB* genes are mapped in upregulated regions. Functional classification of **(B)** LuxS-upregulated DEGs and **(C)** LuxS-downregulated DEGs based on transcriptomic profiling. The green, red, and blue bars represent DEGs classified as molecular function, biological process, and cellular component categories, respectively.

To elucidate the regulatory role of LuxS in *H*. *pylori*, transcriptomic analysis was performed on wild type and Δ*luxS* mutant strains cultured in Brucella broth for 8 h with an initial OD_600_ of 0.2. The raw RNA-seq data were deposited in the NCBI SRA database (BioProject: PRJNA603208). Comparative analysis revealed 187 differentially expressed genes (DEGs) between the wild type and Δ*luxS* mutant strains (Figure 1A), suggesting that LuxS affects the expression of numerous genes. Among them, 122 DEGs were upregulated in the wild type strain, while 65 DEGs were downregulated (listed in Table 1). Gene ontology (GO) analysis indicated that DEGs upregulated by LuxS were mainly involved in establishment of localization, aromatic compound biosynthetic process, peptide transport, cilium or flagellum-dependent cell motility, and amine metabolic process (Figure 1B). LuxS-downregulated genes were mainly involved in oxidation reduction activity, drug metabolic process, amino acid metabolic process, and ATP metabolic process (Figure 1C). We have also performed qPCR to confirmed the expression of these genes, same conclusion is obtained (listed in Supplementary Table 2).

**Table 1:** Differentially expressed genes (DEGs) characterized by RNA-sequencing (Wild type/Δ*luxS*).

**Gene ID**	**Gene name or function**	**Log_**2**_FoldChange**
HP1322	Hypothetical protein	−6.0054
HP0507	NUDIX hydrolase	−2.1722
HP0653	Non-heme iron-containing ferritin	−2.0668
HP0070	Urease accessory protein UreE	−1.9742
HP0902	Hypothetical protein	−1.7708
HP0997	IS605 family transposase TnpB	−1.7683
HP0062	Hypothetical protein	−1.6629
HP0656	Dehypoxanthine futalosine cyclase	−1.6591
HP1334	Hypothetical protein	−1.6398
HP1006	Conjugal transfer protein TraG	−1.6315
HP0901	Membrane protein	−1.6004
HP1570	3-deoxy-D-manno-octulosonate 8-phosphate phosphatase	−1.5416
HP0090	Malonyl CoA-ACP transacylase	−1.5314
HP0307	AtpZ/AtpI family protein	−1.5241
HP1021	Chemotaxis protein CheY	−1.4661
HPt17	tRNA-Arg	−1.4529
HP0944	Aminoacrylate peracid reductase RutC family protein	−1.4398
HP0167	Hypothetical protein	−1.4216
HP0132	L-serine deaminase SdaA	−1.4147
HP0191	Fumarate reductase iron-sulfur subunit	−1.3888
HP1452	tRNA modification GTPase TrmE	−1.3687
HP0060	Hypothetical protein	−1.3398
HP0160	Beta-lactamase HcpD	−1.3271
HP1209	Pseudogene	−1.325
HP1095	IS605 transposase TnpB	−1.3236
HP1131	ATP synthase subunit epsilon	−1.2961
HP1461	Cytochrome c551 peroxidase	−1.2931
HP1291	Thiamine pyrophosphokinase	−1.2902
HP1109	Pyruvate flavodoxin oxidoreductase subunit delta	−1.2644
HP1218	Phosphoribosylamine–glycine ligase	−1.2631
HP1060	Sec-independent protein translocase protein TatB	−1.2423
HP0388	tRNA methyltransferase	−1.2253
HP0143	Pseudogene	−1.2132
HP1243	Membrane protein	−1.2131
HP0221	Nitrogen fixation protein NifU	−1.1862
HP0581	Dihydroorotase	−1.1612
HP0089	Aminodeoxyfutalosine nucleosidase	−1.1585
HP0631	Ni/Fe hydrogenase small subunit	−1.1557
HP1554	30S ribosomal protein S2	−1.1531
HP0133	Serine transporter SdaC	−1.1138
HP0571	Membrane protein	−1.1081
HP0389	Iron-dependent superoxide dismutase	−1.0979
HP0356	Hypothetical protein	−1.0939
HP1524	Lipoprotein	−1.0914
HP1127	Pseudogene	−1.0787
HP1564	ABC transporter substrate-binding protein	−1.071
HP0548	Pseudogene	−1.0657
HP1298	Translation initiation factor IF-1	−1.0634
HP0721	Hypothetical protein	−1.057
HP1016	CDP-diacylglycerol–glycerol-3-phosphate 3-phosphatidyltransferase	−1.0534
HP0873	Membrane protein	−1.0511
HP0975	Aspartyl/glutamyl-tRNA amidotransferase subunit C	−1.05
HP1474	Thymidylate kinase	−1.0494
HP0635	Hydrogenase biosynthesis protein HydE	−1.0447
HP0096	2-hydroxyacid dehydrogenase	−1.0411
HP0632	Ni/Fe hydrogenase large subunit	−1.0367
HP0856	Hypothetical protein	−1.0342
HP0933	6-carboxy-5,6,7,8-tetrahydropterin synthase	−1.0314
HP0780	Hypothetical protein	−1.0306
HP0126	50S ribosomal protein L20	−1.0167
HP1290	Nicotinamide mononucleotide transporter PnuC	−1.0167
HP0013	ATP-binding protein	−1.0159
HP1502	Membrane protein	−1.0126
HP0892	Addiction module toxin	−1.0024
HP0250	ABC transporter ATP-binding protein	1.0096
HP0264	Chaperone protein ClpB	1.0106
HP0454	Hypothetical protein	1.0127
HP1412	Hypothetical protein	1.0176
HP1364	Histidine kinase sensor protein	1.0197
HP1118	Gamma-glutamyltranspeptidase	1.0198
HP1010	Polyphosphate kinase	1.0223
HP0415	Mechanosensitive ion channel protein	1.0253
HP0810	16S rRNA (guanine(966)-N(2))-methyltransferase RsmD	1.0261
HP0606	Membrane protein	1.0341
HP0453	Hypothetical protein	1.0675
HP0431	Protein phosphatase 2C	1.068
HP1499	Restriction endonuclease	1.0721
HP0295	Flagellar hook-associated protein FlgL	1.0729
HP1048	Translation initiation factor IF-2	1.0746
HP0483	Pseudogene	1.0756
HP1047	Ribosome-binding factor A	1.0809
HP0428	Hypothetical protein	1.0815
HP0022	Lipid A phosphoethanolamine transferase	1.0869
HP0880	Hypothetical protein	1.0885
HP1283	Hypothetical protein	1.091
HP1589	Hypothetical protein	1.0985
HP0430	Hypothetical protein	1.1001
HP0985	Hypothetical protein	1.1058
HP0681	Membrane protein	1.1123
HP1049	hypothetical protein	1.1299
HPt36	tRNA-Phe	1.139
HP0915	Pseudogene	1.1429
HP1279	Bifunctional indole-3-glycerol phosphate synthase/phosphoribosylanthranilate isomerase	1.152
HP0806	Metalloprotease	1.1853
HP1390	Hypothetical protein	1.189
HP0885	Lipid II flippase MurJ	1.2119
HP1050	Homoserine kinase	1.2136
HP0815	Flagellar motor protein MotA	1.2218
HP1036	7,8-dihydro-6-hydroxymethylpterin-pyrophosphokinase	1.2375
HP1229	Aspartokinase	1.2479
HP0445	Pseudogene	1.2510
HP0251	ABC transporter permease	1.2716
HP0605	Membrane protein	1.2728
HP1400	Iron(III) dicitrate transport protein FecA	1.2869
HP1238	Formamidase	1.2907
HP0186	Hypothetical protein	1.2945
HP0753	Flagellar biosynthesis protein FliS	1.3035
HP0455	Pseudogene	1.3066
HP0607	Multidrug resistance protein	1.3128
HP0808	Holo-[acyl-carrier-protein] synthase	1.3146
HP0939	Amino acid ABC transporter permease	1.3303
HP1166	Glucose-6-phosphate isomerase	1.3313
HPt08	tRNA-Asn	1.3372
HP0505	Pseudogene	1.3434
HP0816	Flagellar motor protein MotB	1.3505
HP0472	Membrane protein	1.3556
HP0986	Hypothetical protein	1.3571
HP1122	Hypothetical protein	1.3571
HP1284	Heptosyltransferase	1.3574
HP1187	DUF874 family protein	1.3714
HP0423	Hypothetical protein	1.3736
HP0072	Urease subunit beta	1.3741
HP0447	Hypothetical protein	1.3852
HP0722	Membrane protein	1.3852
HP1148	tRNA (guanine-N(1)-)-methyltransferase	1.3861
HP1473	Amidophosphoribosyltransferase	1.3894
HP0903	Acetate kinase	1.3919
HP0759	Membrane protein	1.3932
HP1233	Hypothetical protein	1.4047
HP0168	Tetratricopeptide repeat protein	1.4140
HP1584	tRNA N6-adenosine threonylcarbamoyltransferase	1.4356
HP0640	tRNA nucleotidyltransferase/poly(A) polymerase	1.4423
HP1028	Hypothetical protein	1.4482
HP0757	N-carbamoylputrescine amidase	1.4625
HPt26	tRNA-Pro	1.471
HP0758	Sodium:proton antiporter	1.4774
HP0434	Pseudogene	1.4937
HP0937	Pseudogene	1.4976
HP1556	Cell division protein FtsI	1.5018
HP0754	5-formyltetrahydrofolate cyclo-ligase	1.5228
HP0073	Urease subunit alpha	1.5257
HP1082	Multidrug ABC transporter ATP-binding protein/permease	1.5375
HP1582	Pyridoxine 5'-phosphate synthase	1.546
HP1583	4-hydroxythreonine-4-phosphate dehydrogenase	1.571
HP1000	Chromosome partitioning ATPase	1.5789
HP0346	Pseudogene	1.6463
HP0677	Membrane protein	1.6504
HP1440	Hypothetical protein	1.6506
HP0936	Proline/betaine transporter ProP	1.6587
HP0081	Hypothetical protein	1.6803
HP0209	Membrane protein	1.7166
HP1230	DNA replication regulator family protein	1.7243
HP0432	Pseudogene	1.7561
HP1076	Hypothetical protein	1.7595
HP0755	Hypothetical protein	1.7605
HP0131	Hypothetical protein	1.7766
HPt20	tRNA-Met	1.7913
HP0686	Iron(III) dicitrate transport protein FecA	1.8017
HPt01	tRNA-Glu	1.8654
HP0107	Cysteine synthetase CysK	1.8681
HP0714	RNA polymerase factor sigma-54	1.869
HP0715	ABC transporter ATP-binding protein	1.9132
HP0039	Pseudogene	1.9381
HP1080	Restriction endonuclease	1.9476
HP1051	Hypothetical protein	1.9653
HP0998	IS605 family transposase TnpA	1.9908
HP0603	Hypothetical protein	2.0065
HP0682	Hypothetical protein	2.0177
HP0846	Type I restriction-modification system endonuclease	2.0194
HP0676	Methylated-DNA–protein-cysteine methyltransferase	2.1394
HP0343	Pseudogene	2.1668
HP0602	3-methyladenine DNA glycosylase	2.1685
HP0339	Lysozyme	2.2347
HP0456	Hypothetical protein	2.2801
HP0342	Hypothetical protein	2.2862
HP0471	Potassium transporter	2.3209
HP0340	Hypothetical protein	2.3228
HP0448	Hypothetical protein	2.3457
HP0752	Flagellar hook-associated protein FliD	2.349
HP1121	Cytosine specific DNA methyltransferase	2.36
HP0751	Flagellar protein FlaG	2.3686
HP0876	Outer membrane protein	2.3998
HP1081	Hypothetical protein	2.5851
HP1119	Flagellar hook-associated protein FlgK	2.7713
HP1120	Hypothetical protein	2.9003
HP0807	Iron(III) dicitrate transport protein FecA	2.928
HP0294	Acylamide amidohydrolase	3.9658

### AI-2 Induces UreAB Expression and Bacterial Urease Activity in *H*. *pylori*

**Figure 2:**
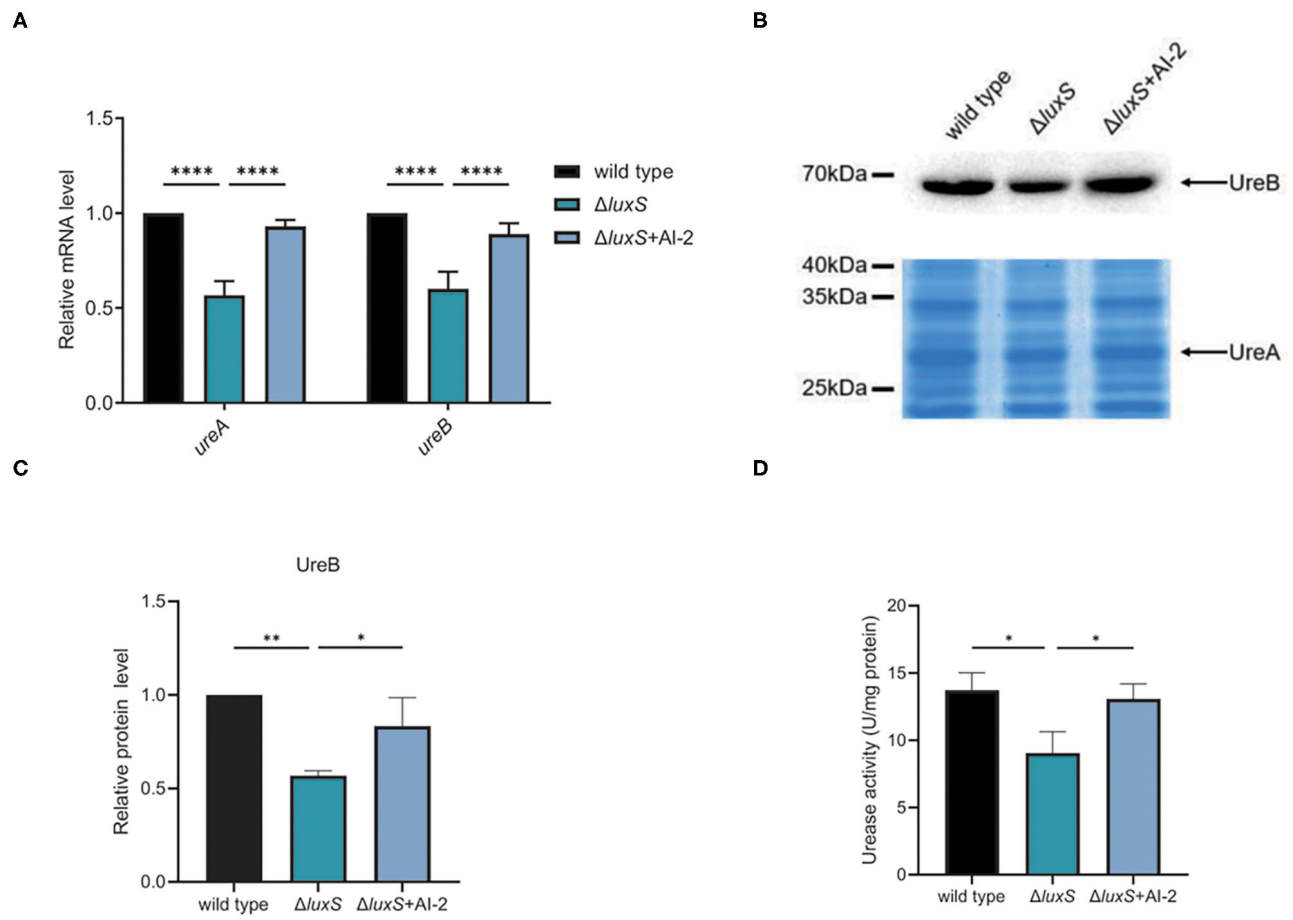
AI-2 regulated the expression of urease. **(A)** Transcriptional levels of UreA and UreB regulated by AI-2, quantified by qPCR. Relative mRNA levels represent mRNA levels of UreA and UreB in the mutant strains normalized to those in the wild type strain. **(B)** Protein levels of UreA and UreB regulated by AI-2. Expression of UreB was determined by western blotting, with the arrow indicating protein bands of UreB. Expression of UreA were detected by SDS-PAGE followed by staining with Coomassie brilliant blue, with the arrow indicating protein bands of UreA. **(C)** Densitometric analysis of UreB protein bands. Total protein bands stained with Coomassie brilliant blue were used as loading control. **(D)** Determination of urease activity in cell lysates. Values are shown as averages of three biological independent experiments and bars represent standard deviations. ****P < 0.0001, **P < 0.01, *P < 0.05.

From the transcriptomic analysis, we determined that UreA and UreB expression was lower in the Δ*luxS* mutant than in the wild type strain (Table 1), suggesting that quorum sensing might be important for acid acclimation in *H*. *pylori*. To confirm this hypothesis, we first investigated the expression of UreA and UreB in the wild type and Δ*luxS* mutant strains by qPCR (Figure 2A). The Δ*luxS* mutant strain displayed only half the mRNA expression levels of UreA and UreB as in the wild type strain, which concurred with the results of the transcriptomic analysis. We then complemented LuxS by introducing a *luxS* coding sequence downstream of the *ureAB* promoter region and verified UreA and UreB expression. The results demonstrated that LuxS complementation restored UreA and UreB expression levels in the Δ*luxS* mutant to those in the wild type strain (Supplementary Figure 1). Since LuxS is involved in both quorum sensing as an AI-2 synthase and in non-quorum sensing as part of the activated methyl cycle, we hypothesized that AI-2 might be responsible for activation of urease expression. To examine this possibility, chemical complementation of LuxS was performed by supplementing the Δ*luxS* mutant with AI-2 (Δ*luxS*+AI-2). The results revealed that supplementation with AI-2 restored UreA and UreB expression levels in the Δ*luxS* mutant to those in the wild type strain (Figure 2A), suggesting that LuxS induced urease expression through AI-2. We also performed western blotting to investigate the UreB expression regulated by AI-2, we found that protein level of UreB in Δ*luxS* was approximately half of the levels in wild type and Δ*luxS*+AI-2 (Figures 2B,C). To ensure that the decreased UreAB expression might lead to a lower bacterial urease activity, we harvested the cell lysates, and measured bacterial urease activity. The result showed that the urease activity was lower in Δ*luxS* compared to wild type and Δ*luxS*+AI-2 (Figure 2D). These results suggested that AI-2 induced the expression of UreAB, leading to an upregulated urease activity in *H*. *pylori*.

### AI-2 Downregulates Expression of Transcriptional Regulator HP1021, Which Depresses Urease Expression

**Figure 3:**
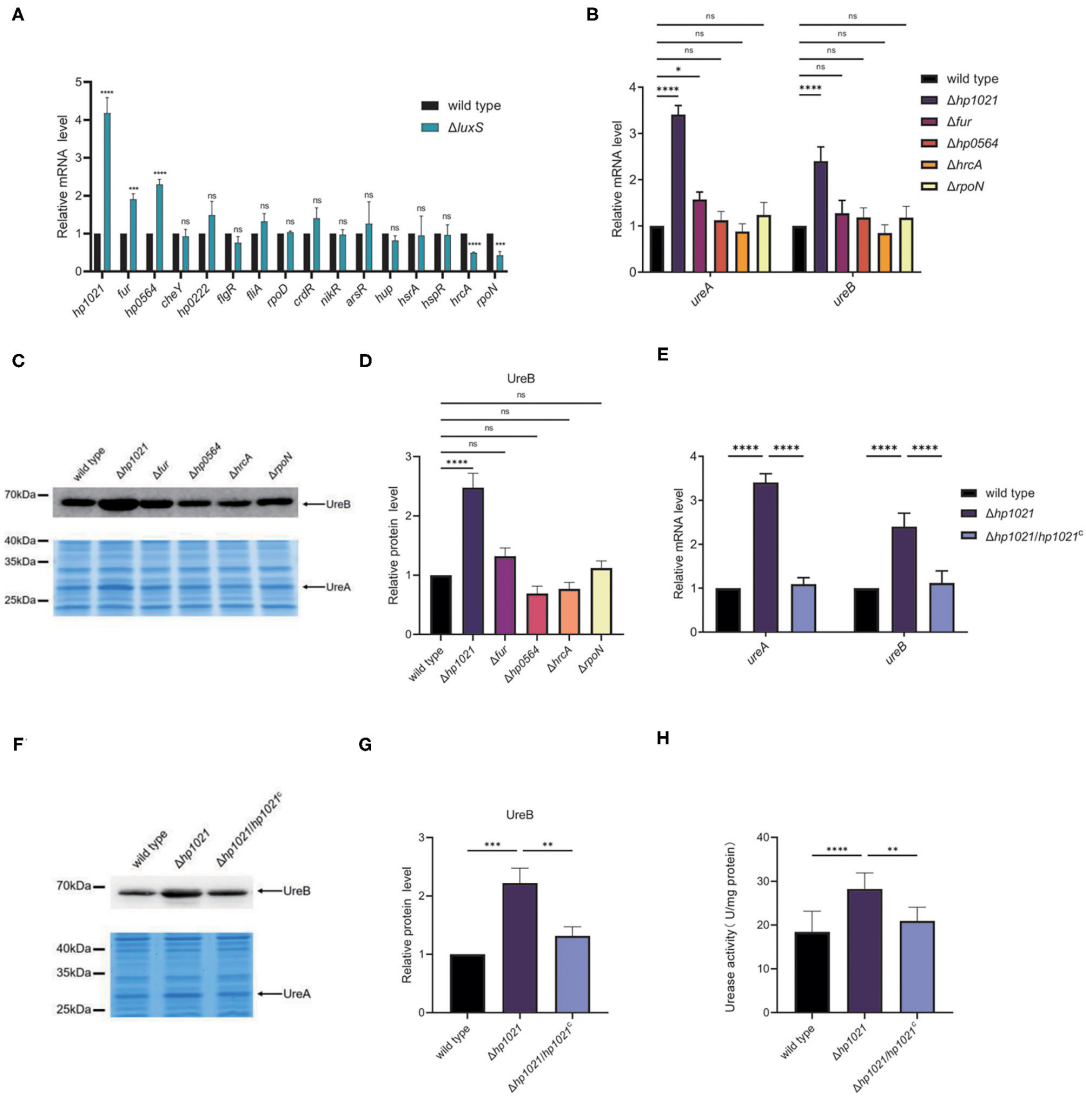
HP1021 inhibited urease expression while AI-2 downregulated HP1021 expression. **(A)** The mRNA expression levels of transcriptional regulators determined by qPCR. **(B)** The mRNA expression levels of UreA and UreB regulated by transcriptional regulators, as determined by qPCR. Relative RNA levels represent the mRNA levels in the mutant strains normalized to those in the wild type strain. Values represent averages of three biological independent experiments and bars represent standard deviations. **(C)** Expression of UreA and UreB proteins regulated by transcriptional regulators. UreB expression was detected by Western blotting, with the arrow indicating UreB protein bands. Expression of UreA was detected by SDS-PAGE followed by staining with Coomassie brilliant blue. The arrow indicates UreA protein bands. **(D)** Densitometric analysis of UreB protein bands. Total protein bands stained with Coomassie brilliant blue were used as loading control. **(E)** The mRNA expression of UreA and UreB regulated by HP1021. **(F)** Expression of UreA and UreB proteins regulated by HP1021. **(G)** Densitometric analysis of UreB protein bands. Total protein bands stained with Coomassie brilliant blue were used as loading control. **(H)** Determination of urease activity in cell lysates. Values represent averages of three biological independent experiments and bars represent standard deviations. ****P < 0.0001, ***P < 0.001, **P < 0.01, *P < 0.05, ns, non-significant.

Since AI-2 promoted the transcription of *ureAB*, we suspected that AI-2 might induce the expression of urease by regulating the expression of transcriptional regulators. *H. pylori* strain 26695 harbors 16 transcriptional regulators (54), which we examined in our transcriptomic analysis (Supplementary Table 3) and confirmed by qPCR (Figure 3A). The expression levels of HrcA (HP0111) and RpoN (HP0714) were lower in the Δ*luxS* mutant strain than those in the wild type strain, whereas those of HP1021, Fur (HP1027), and HP0564 were higher. We then determined which transcriptional regulator might be involved in the regulation of urease expression. We constructed Δ*hp1021*, Δ*fur*, Δ*rpoN*, Δ*hrcA*, and Δ*hp0564* mutant strains, and investigated the expression of UreA and UreB in the mutant and wild type strains (Figures 3B–D). Compared to the wild type strain, the expression of UreA and UreB was similar at the RNA and protein levels in the Δ*fur*, Δ*rpoN*, Δ*hrcA*, and Δ*hp0564* mutant strains, suggesting that Fur, RpoN, HrcA, and HP0564 were not involved in the regulation of urease expression. However, the mRNA expression levels of UreA and UreB were 3.5 and 2.5 times higher while the protein level of UreB was 2.4 times higher in the Δ*hp1021* mutant strain than in the wild type strain, respectively. We also performed chromosomal complementation of HP1021 in the Δ*hp1021* mutant strain (Δ*hp1021*/*hp*1021^C^), revealing that complementation with HP1021 restored the expression levels of UreA and UreB to those in the wild type strain (Figures 3E–G). The urease activity was confirmed, and were consistent with the higher urease activity in the Δ*hp1021* mutant strain compared to wild type strain and Δ*hp1021*/*hp*1021^C^ (Figure 3H). These results demonstrated that HP1021 represses the expression of urease, leading to decreased bacterial urease activity.

### AI-2 Induced Urease Expression Through HP1021

**Figure 4:**
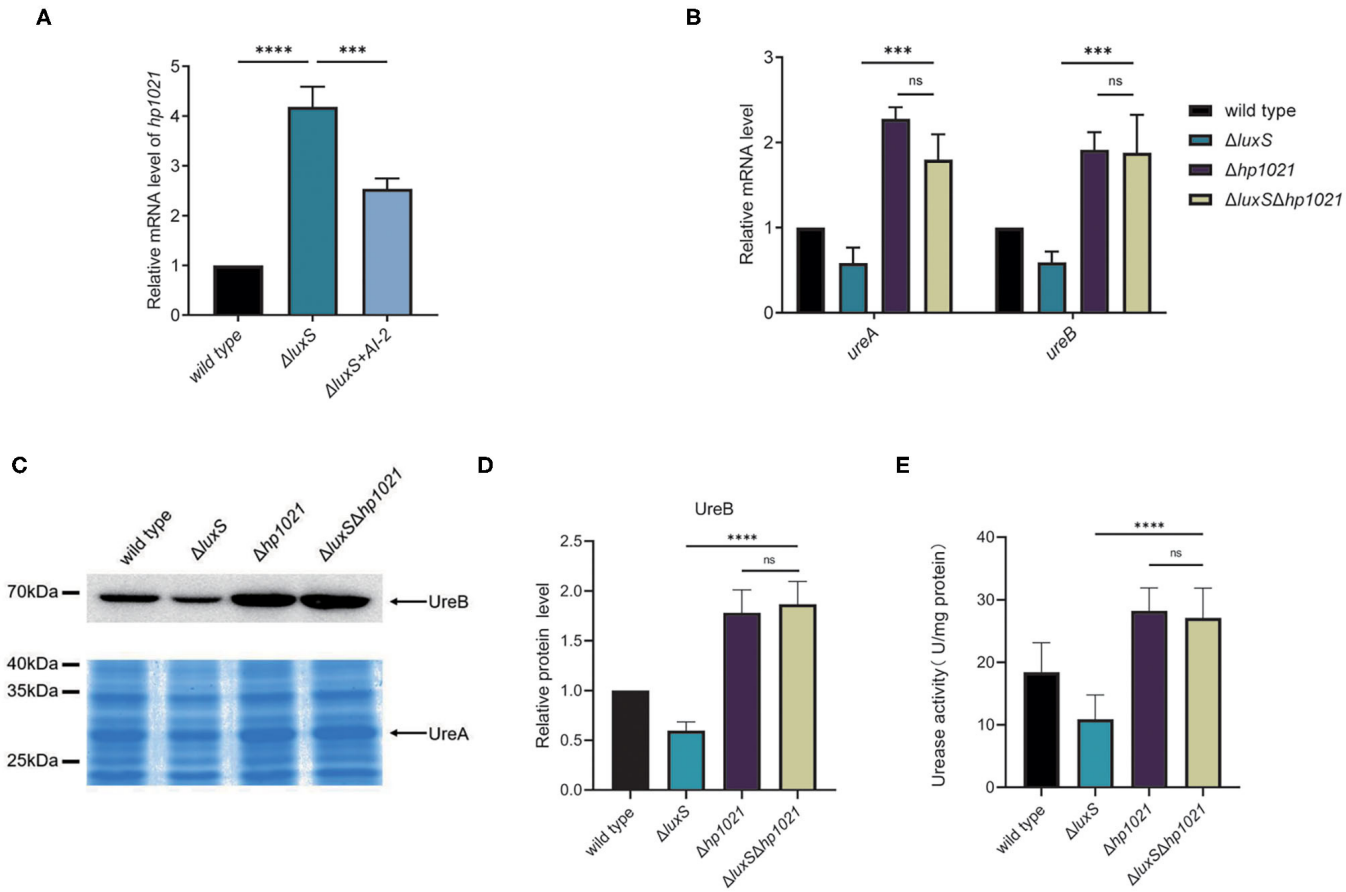
LuxS induced the expression of urease through HP1021. **(A)** mRNA expression levels of HP1021 regulated by LuxS and supplemented with AI-2, measured by qPCR. Relative mRNA levels represent mRNA levels of HP1021 in the mutant strain and mutant strain supplemented with AI-2 normalized to those in the wild type strain. **(B)** mRNA expression of UreA and UreB regulated by LuxS and HP1021. Relative mRNA levels represent mRNA levels of UreA and UreB in the mutant strains normalized to those in the wild type strain. **(C)** Expression of UreA and UreB proteins regulated by LuxS and HP1021. UreB expression was detected by western blotting, and protein bands are indicated with the arrow. UreA expression was measured by SDS-PAGE gel followed by staining with Coomassie brilliant blue. The arrow indicates UreA protein bands. **(D)** Densitometric analysis of UreB protein bands. Total protein bands stained with Coomassie brilliant blue were used as loading control. **(E)** Determination of urease activity in cell lysates. Values are the averages from three biological independent experiments and bars represent standard deviations. ****P < 0.0001, ***P < 0.001, ns, non-significant.

Next, we investigated whether HP1021 was responsible for the induction of urease expression by AI-2. We first confirmed the expression of HP1021 in the wild type and Δ*luxS* mutant strains supplemented with or without AI-2. AI-2 supplementation resulted in decreased HP1021 expression, suggesting that HP1021 expression was downregulated by AI-2 (Figure 4A). Complementation of LuxS also restored the HP1021 expression in Δ*luxS* to the level similar with that in wild type strain (Supplementary Figure 1). Since HP1021 was the only transcriptional regulator regulated by AI-2 while manipulating the expression of urease, we investigated whether HP1021 was responsible for the regulation of urease expression by AI-2. For this purpose, we constructed the Δ*luxS*Δ*hp1021* mutant strain and compared the expression of UreA and UreB in the wild type and Δ*luxS*, Δ*hp1021*, and Δ*luxS*Δ*hp1021* mutant strains (Figure 4B). The mRNA expression levels of UreA and UreB were similar in the Δ*hp1021* and Δ*luxS*Δ*hp1021* mutant strains, but higher than in the Δ*luxS* mutant strain, suggesting that HP1021 lies downstream of LuxS and plays an indispensable role in the regulation of urease expression by AI-2. We also confirmed these results at the protein level through western blotting and SDS-PAGE, and measured bacterial urease activity, supporting the conclusion that AI-2 induced urease expression and urease activity through HP1021 (Figures 4C–E).

### HP1021 Directly Binds to the Promoter Region of *ureAB*

**Figure 5:**
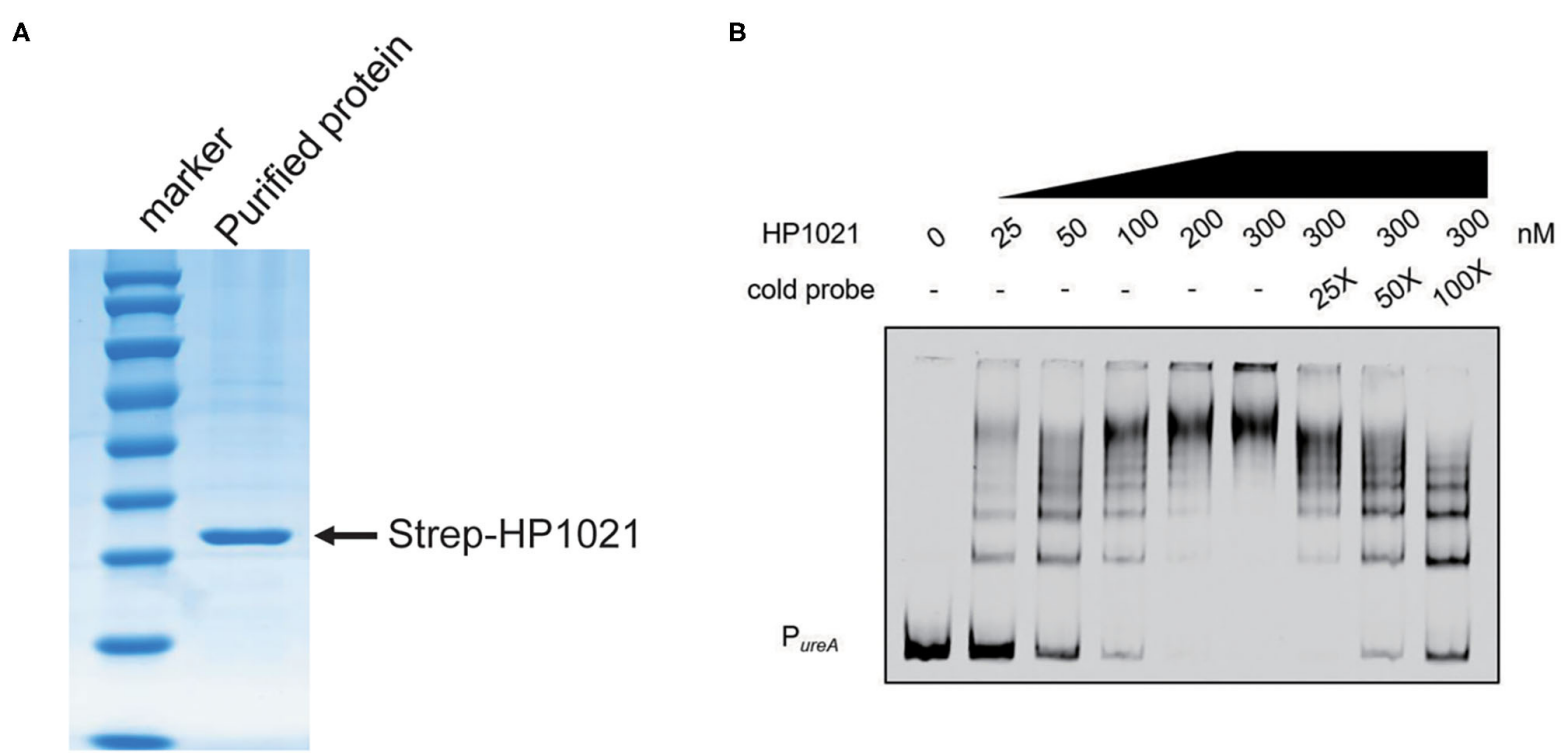
HP1021 bound directly to the *ureA* promoter region. **(A)** Coomassie-stained gel of purified Strep-tagged HP1021 protein, indicated by the arrow. **(B)** Electrophoretic mobility shift assay (EMSA) results of the interaction between HP1021 and the *ureAB* promoter. Fragments of the 300-bp *ureAB* promoter region labeled at the 5'-end with AF700 were incubated with increasing concentrations of Strep-HP1021 in the presence or absence of unlabeled *ureAB* promoter (cold probe). The arrow indicates bands representing 5' AF700-labeled *ureAB* probe bound by HP1021.

HP1021 is known as an atypical orphan response regulator involved in the initiation of DNA replication in *H. pylori* by directly binding to the promoter region of *oriC* (55, 56). Therefore, we investigated whether HP1021 repressed urease expression by directly binding to the promoter region of *ureAB*. The expression of Strep-tagged HP1021 was induced through a TetR induction system and HP1021 was purified by Strep-tag affinity chromatography (Figure 5A). Promoter fragments of *ureAB* with 5'-labeled AF700 were then incubated with increasing amounts of purified HP1021. The results demonstrated that the DNA probes were fully bound by HP1021 when the concentration of HP1021 was increased to 200 nM (Figure 5B). To verify the specific affinity between HP1021 and the *ureAB* promoter fragments, excess non-labeled probe was used as a cold probe, with the results indicating that the cold probe relieved the AF700-labeled P*ureA* bound by HP1021 (Figure 5B). These results suggested that HP1021 had a high binding affinity to the promoter region of *ureAB*, resulting in the repression of *ureAB* transcription.

### Identification of HP1021 Binding Site on *ureAB* Promoter Region

**Figure 6:**
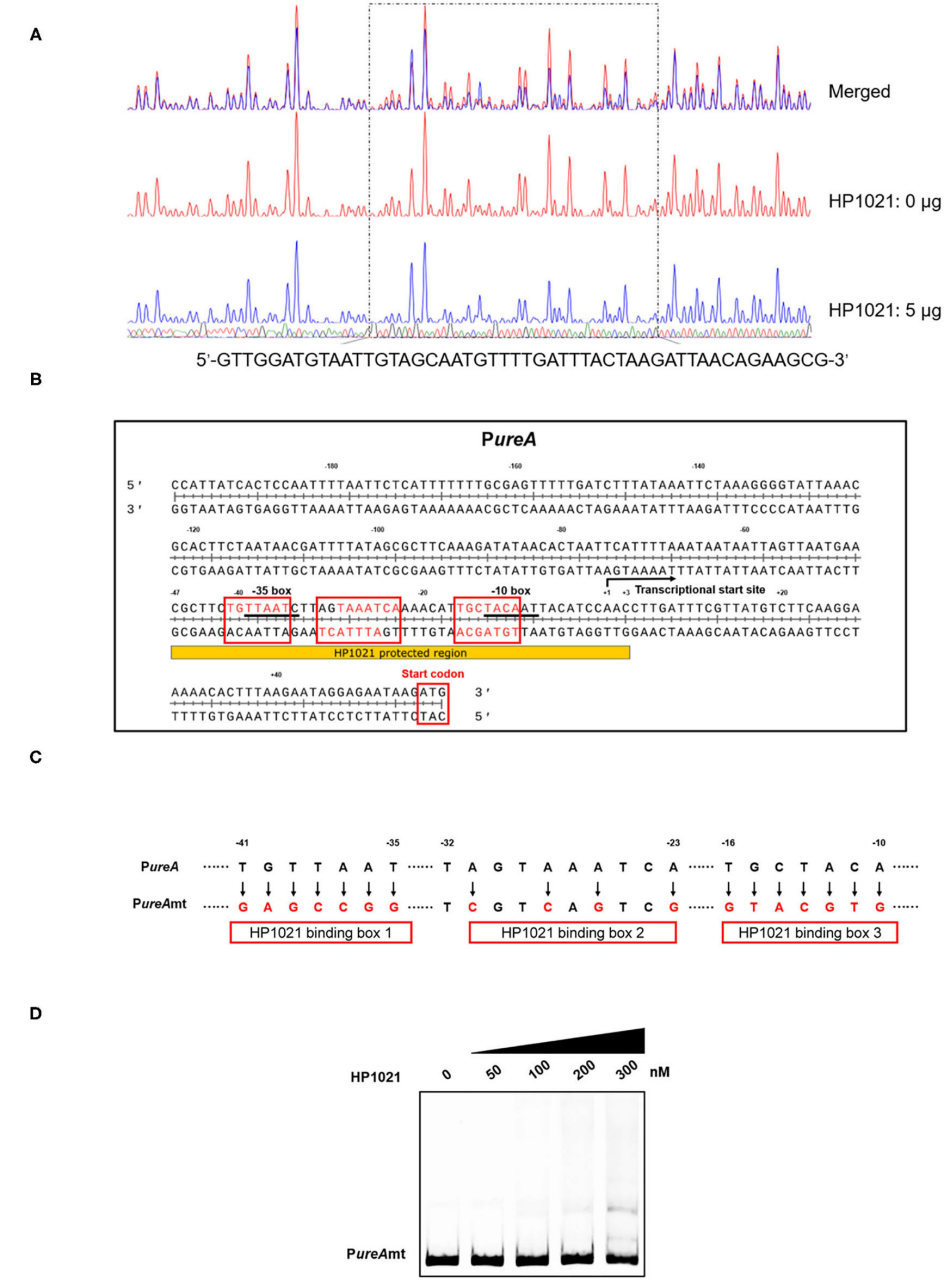
Determination of the binding site of HP1021 on the *ureAB* promoter region. **(A)** Dye primer-based DNase I footprinting assay of HP1021 and the *ureAB* promoter region. Protected fluorograms were obtained by incubating fragments of the *ureAB* promoter region with or without HP1021. The nucleotide sequence of the protected region is shown below. **(B)** Analysis of the HP1021 binding site and consensus HP1021 binding boxes. Sequence shown is P*ureA* with numbers represent the position related to the transcriptional start site. The consensus −10 and −35 box are underlined, while the HP1021 protected sequence is indicated. The transcriptional start site was indicated with arrow; three HP1021 binding boxes were indicated with red box, while the putative consensus HP1021 binding sequences within were shown with red. **(C)** Mutagenesis of the HP1021 binding site. The replaced nucleotides are indicated with arrows and the mutation probe (P*ureA*mt) is shown. **(D)** Electrophoretic mobility shift assay (EMSA) results of the interaction between HP1021 and P*ureA*mt probe. P*ureA*mt labeled at the 5'-end with AF700 was incubated with increasing concentrations of Strep-HP1021 protein. The arrow indicates P*ureA*mt probe bands.

To elucidate the molecular mechanism by which HP1021 repressed urease expression, we performed a DNase I footprinting assay using dye primer sequencing to identify the binding site of HP1021 on the promoter region of *ureAB* (Figure 6A). The results demonstrated that HP1021 protected the promoter region of *ureAB* (5'-CGCTTCTGTTAATCTTAGTAAATCAAAACATTGCTACAATTACATCCAAC-3'), ranging from −47 to +3 with respect to the transcriptional start site, as previously reported (52), overlapping the −10 and −35 consensus sequences (Figure 6B). The HP1021 protected DNA sequence revealed three HP1021 binding boxes harboring five putative reported consensus HP1021 binding sequence in sense and antisense strands (57). These results suggested that multiple HP1021 proteins bound to the promoter region of *ureAB*, strongly inhibiting *ureAB* transcription by preventing RNA polymerase binding. We then prepared a mutant probe (P*ureA*mt) through site-directed mutagenesis to replace nucleotides on the P*ureA* (Figure 6C), and performed EMSA to investigate the interaction between HP1021 and P*ureA*mt (Figure 6D). The results indicated that HP1021 did not interact with P*ureA*mt, suggesting that the putative HP1021 binding motifs were critical to the interaction between HP1021 and the promoter region of *ureAB*.

## Discussion

*H. pylori* is a human pathogen that colonizes the stomach and can cause chronic infection, leading to the occurrence and development of gastric diseases such as gastric cancer (1, 2). More than 50% of the world's population is infected with *H. pylori*, and people tend to carry this pathogen without intervention. Indeed, ~80–90% of infected individuals exhibit no significant clinical symptoms (58). These findings suggest that *H*. *pylori* has successfully adapted to the gastric environment and is capable of avoiding the host immune defense system. However, the disease outcomes of *H*. *pylori* infection are strongly associated with factors such as host genetics, the environment, and diet; therefore, understanding the pathogenesis of *H. pylori* necessitates elucidating the mechanisms that regulate its virulence.

Urease plays a key role in acid acclimation for *H*. *pylori*, as it hydrolyzes intracellular urea to ammonia and carbon dioxide, buffering the cytoplasm under the acidic environment (59). Urease expression is abundant in *H. pylori*, but it varies in different environments. Urease expression can be directly or indirectly activated through the ArsRS two-component system in response to external acidic pH (26–28), and also by intracellular nickel concentrations through NikR (60). The results of these studies suggest that higher expression of urease is required under low pH and high nickel conditions. In the current study, we determined that the expression of urease was induced by the quorum-sensing signal molecule AI-2 (Figure 2). AI-2 reportedly activates the expression of flagella to increase the motility of *H. pylori*, thus enabling cells to disperse to other sites when bacterial density increases (41, 42, 61–64). Dispersal of bacteria is important for the survival of *H. pylori* since nutrients and space become limited when bacterial density increases. Previous studies reported that AI-2 also inhibits biofilm formation and promotes bacterial dispersal (44, 65). Therefore, we suspect that when AI-2 activates bacterial movement at high cell density, it also upregulates urease expression to combat acidic gastric juice.

In this study, we found that Fur, HP0564, HrcA, and RpoN were not involved in the regulation of urease expression (Figures 3B,C). Fur is a transcriptional regulator involved in iron homeostasis, suggesting that iron is not involved in the regulation of urease expression in *H. pylori*, which concurs with previously reported results that iron has no effect on urease expression (66). HP0564 is a transcriptional regulator with an unknown regulon, HrcA is involved in heat shock response, and RpoN is a sigma factor that is responsive to nutrient stress and regulates flagellar expression (67–69). However, other transcriptional regulators involved in urease expression warrant further investigation, as other environmental conditions might alter urease expression. Urease regulation has been studied in other bacteria, including by carbon storage regulator (CsrA) in *Yersinia pseudotuberculosis* and through Fur in *Helicobacter hepaticus* (70, 71). These results suggest that nutrient conditions are closely related to urease expression. Studies have shown that urease expression is not only involved in initial colonization, but is also critical for maintaining chronic infection (72). Therefore, the expression of urease is likely critical for the pathogenesis of *H. pylori*, although the environmental factors involved in its regulation need to be further explored.

Quorum sensing plays an important role in the regulation of bacterial behavior, such as the expression of virulence factors (73). The transcriptomic study indicated that LuxS represses the expression hydrogenase (HP0631-0632, HP0635) and iron-dependent superoxide dismutase (SodB), suggesting that LuxS is involved in iron related cellular activity. Expression of flagellar related genes including FlgL, MotA, FliS, MotB, FliD, FlaG and FlgK are also activated by LuxS (Supplementary Table 2), suggesting that LuxS not only activated the flagellar formation, but also controls movement of *H*. *pylori*. We also noticed that multidrug efflux pump components HP0607 and HP1082 are also repressed in Δ*luxS*, suggesting that quorum sensing might be also associated with drug resistance in *H*. *pylori*. These results suggest that LuxS plays important roles in regulating the growth and pathogenesis of *H*. *pylori*.

The molecular mechanism of quorum sensing has been studied in various pathogens, including *Vibrio cholerae* and *Pseudomonas aeruginosa*. In general, quorum sensing systems consist of autoinducer synthases, autoinducer signals, and response regulators that alter gene expression (74). In *V*. *cholerae*, the transcriptional regulator LuxO activates autoinducer signaling through the transcription of four quorum-sensing regulatory sRNAs, which regulates the quorum-sensing master regulator HapR (75, 76). In *H. pylori*, we found that HP1021 is a transcriptional regulator that responds to AI-2, and to the best of our knowledge, HP1021 is the first transcriptional regulator identified in *H*. *pylori* that plays an important role in quorum sensing regulation. HP1021 reportedly regulates the expression of numerous genes, including genes involved in Fe-S assembly, acetone metabolism, DNA replication initiation, and virulence gene *cagA* (56, 57). Quorum sensing in *H*. *pylori* might regulate these processes through HP1021 repressed by AI-2. In this study, we found that AI-2 cannot restore the expression of HP1021 in Δ*luxS* to the level similar to wild type (Figure 4), this might attribute to the concentration of exogenous supplementation of AI-2 decreased followed by bacterial growth, resulted in decreased AI-2 function as discussed in our previous work (46). However, according to our results, AI-2 is sufficient to restore urease expression in Δ*luxS* through HP1021 (Figure 2). In this study, we focused on the molecular mechanism by which AI-2 regulates urease expression, but the roles of other transcriptional regulators, such as Fur, RpoN, HrcA, and HP0564, remain to be elucidated.

Our study findings indicated that HP1021 is a negative regulator of urease expression by directly binding to the promoter region of *ureAB*. To our knowledge, HP1021 is the first transcriptional regulator that can directly exert a repressive effect on urease structural genes' expression in *H*. *pylori*. The EMSA results demonstrated that the probes were fully bound in the presence of 200 nM Strep-HP1021 (Figure 5B), suggesting high binding affinity between HP1021 and the *ureA* promoter. We also observed multiple band shifts, indicating that HP1021 bound to multiple sites on the *ureA* promoter. Studies have reported multiple band shifts for the binding of HP1021 to the promoter regions of *oriC, hyuA*, and *katA*, with 2–3 consensus HP1021 binding boxes identified on the probe fragments, while single band shifts were observed for *gluP*, as only one HP1021 binding site was found on its promoter region (56, 57). The results of the DNase I footprinting assay indicated that HP1021 protected DNA fragments ranging from −47 to +3 with respect to the transcriptional start site, overlapping the −10 and −35 consensus sequences. This suggests that binding of HP1021 inhibits *ureAB* transcription by preventing RNA polymerase binding. DNA analysis revealed the presence of three HP1021 binding boxes, suggesting that multiple HP1021 molecules bind simultaneously, thus repressing urease expression.

In conclusion, our study findings indicate that AI-2 upregulates urease expression at the mRNA and protein levels through repression of HP1021 in *H*. *pylori*. Further, HP1021 is the first transcriptional regulator identified in *H*. *pylori* that plays a role in quorum sensing circuits. HP1021 directly binds to the promoter region of *ureAB*, resulting in repression of urease expression. Our data suggest that AI-2 might play an important role in facilitating bacterial dispersal to a new niche by upregulation of bacterial urease expression and activity to neutralize intracellular pH when encountering gastric juice.

## Data Availability Statement

The datasets presented in this study can be found in online repositories. The names of the repository/repositories and accession number(s) can be found at: https://www.ncbi.nlm.nih.gov/, PRJNA603208.

## Author Contributions

YW and FS designed the study. HY, XH, and XiaocZ performed the experiments. XX and XiaoyZ analyzed the data. YW and HY wrote the manuscript. All authors have read and approved the submitted version.

## Funding

This work was supported by grants from the National Natural Science Foundation of China (Grant Nos. 81701980 and 82072316), the Natural Science Foundation of Fujian Province, China (Grant Nos. 2019J01295 and 2018J01837), Key Projects of the Natural Science Foundation of Fujian Province, China (Grant No. 2020J02019), and Fujian Medical University Talent Startup Fund (XRCZX2017027, XJ2021011202, and 2017XQ1008).

## Conflict of Interest

The authors declare that the research was conducted in the absence of any commercial or financial relationships that could be construed as a potential conflict of interest.

## Publisher's Note

All claims expressed in this article are solely those of the authors and do not necessarily represent those of their affiliated organizations, or those of the publisher, the editors and the reviewers. Any product that may be evaluated in this article, or claim that may be made by its manufacturer, is not guaranteed or endorsed by the publisher.
