# Supplementary material for: AI-2 Induces Urease Expression Through Downregulation of Orphan Response Regulator HP1021 in Helicobacter pylori
Source: Front Med (Lausanne). 2022 Apr 1;9:790994. doi: 10.3389/fmed.2022.790994 (PMC9010608; doi:10.3389/fmed.2022.790994)
Supplement: Supplementary file 1 [file Image_1.pdf]

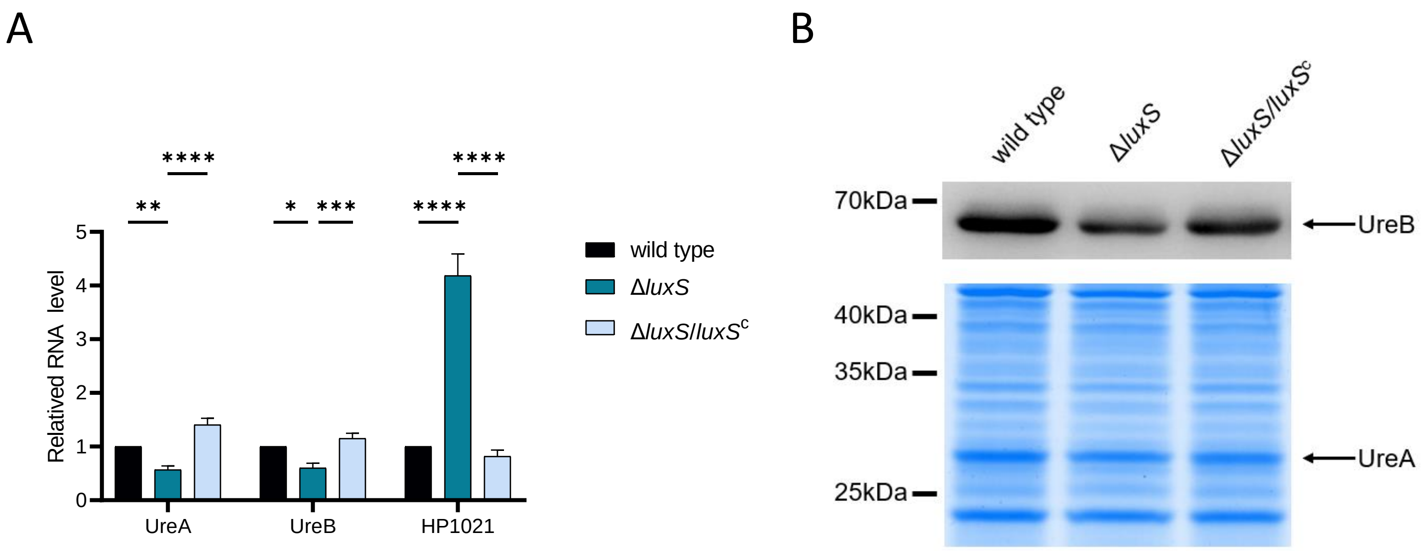

Figure S1. LuxS activated urease expression while repressed HP1021 expression. (A) mRNA expression levels of UreA, UreB and HP1021 regulated by LuxS, determined by qPCR. Relative RNA levels represent the mRNA levels in Δ*luxS* and Δ*luxS/luxS*^{C} strains normalized to those in the wild-type strain. Values represent averages of three independent experiments and bars represent standard deviations. (B) Expression of UreA and UreB proteins regulated by LuxS. UreB expression was detected by western blotting, with the arrow indicating UreB protein bands. Expression of UreA was detected by SDS-PAGE followed by staining with Coomassie blue. The arrow indicates UreA protein bands. Values represent averages of three independent experiments and bars represent standard deviations. ****$P < 0.0001$, ***$P < 0.001$, **$P < 0.01$, *$P < 0.05$, ns, non-significant.
