# Supplementary material for: AI-2 Induces Urease Expression Through Downregulation of Orphan Response Regulator HP1021 in Helicobacter pylori
Source: Front Med (Lausanne). 2022 Apr 1;9:790994. doi: 10.3389/fmed.2022.790994 (PMC9010608; doi:10.3389/fmed.2022.790994)
Supplement: Supplementary file 2 [file Table_1.pdf]

Supplementary Table 1. Primers used in this study

Primers	Sequence (5' to 3')
Construction of *H. pylori* mutants and complemented mutants	
hp1021-up-F	ctatagggcgaattgggtaccTATTATAACGAAGCTTTAGATAGAGAAGCG
hp1021-up-R	cgcccagtATCGTCTTCAATGATTAAGATTTTCATC
hp1021-*aphA3*-F	attgaagacgatACTGGGCGGTTTTATGGACA
hp1021-*aphA3*-R	ggtaagttatatttccggcgAAATTAAAAATGAAGTTTTAGCACGTG
hp1021-down-F	CGCCGGAAATATAACTTACCGC
hp1021-down-R	caggaattcgatatcaagcttAGTTGAAATGAGAACCTCTATTGGAA
fur-up-F	ctatagggcgaattgggtaccTACAATCCTTACGGCCTCATAGC
fur-up-R	aaccgcccagtAATGGATTCCAAAGTTTCTAATCTTTT
fur-*aphA3*-F	gaatccattACTGGGCGGTTTTATGGACA
fur-*aphA3*-R	ctttacaccacacaaacaAAATTAAAAATGAAGTTTTAGCACGTG
fur-down-F	ttTGTTTGTGTGGTGTAAAGAATGCC
fur-down-R	caggaattcgatatcaagcttTGATCGTTTATGAGCCGGTTAGT
hp0564-up-F	ctatagggcgaattgggtaccCCATTGCGTTCCCATAAGGG
hp0564-up-R	accgcccagtCTAATTCCATTAAAATTTCCTTTTTTAGTT
hp0564-*aphA3*-F	atggaattagACTGGGCGGTTTTATGGACA
hp0564-*aphA3*-R	gataccttaaagtaggcttaAAATTAAAAATGAAGTTTTAGCACGTG
hp0564-down-F	TAAGCCTACTTTAAGGTATCAGCTCTTAA
hp0564-down-R	caggaattcgatatcaagcttGCGGCTATTTGATGCATATAGCT
hrcA-up-F	ctatagggcgaattgggtaccTAAGGAGGGCGCGCGTAA
hrcA-up-R	accgcccagtTCTTGATGAAAGAACCCTCGCA

hrcA-aphA3-F	ttcatcaagaACTGGGCGGTTTTATGGACA
hrcA-aphA3-R	gtcaaaaaataaagggctgAAATTAAAAATGAAGTTTTAGCACGTG
hrcA-down-F	tCAGCCCTTTATTTTTTGACATTTTAG
hrcA-down-R	caggaattcgatatcaagcttGATTTTCCCGTTTTCTTTTTCTTC
rpoN-up-F	ctatagggcgaattgggtaccTAAAAAAACCAAAATCGTTTCAGACG
rpoN-up-R	aaaccgcccagtGTAAGATCGCCATGTCTATACCTTAAA
rpoN-aphA3-F	gatcttacACTGGGCGGTTTTATGGACA
rpoN-aphA3-R	cgcgcatcaaatagagccttAAATTAAAAATGAAGTTTTAGCACGTG
rpoN-down-F	AAGGCTCTATTTGATGCGCG
rpoN-down-R	caggaattcgatatcaagcttTGGATTTAACCATTCGCATGG
hp1021-up-F1	ctatagggcgaattgggtaccTATTATAACGAAGCTTTAGATAGAGAAGCG
hp1021-up-R1	tggcggaATCGTCTTCAATGATTAAGATTTTCATC
hp1021-CAT-F	cattgaagacgatTCCGCCATATTGTGTTGAAACA
hp1021-CAT-R	aagttatatttccggcgGGGCACCAATAACTGCCTTAAA
hp1021-down-F1	cccCGCCGGAAATATAACTTACCGC
hp1021-down-R1	caggaattcgatatcaagcttAGTTGAAATGAGAACCTCTATTGGAA
hp1021^{C}-up-F	ctatagggcgaattgggtaccTTCAGTGAAATATTACTTGCAAGATCC
hp1021^{C}-up-R	ggTAATGCAGTATTATGGGGTTAGGGG
hp1021^{C}-P*ureA*-F	ccccataatactgcattaCCATTATCACTCCAATTTTAATTCTCA
hp1021^{C}-P*ureA*-R	agattttcatCTTATTCTCCTATTCTTAAAGTGTTTTTCC
hp1021^{C}-F	ggagaataagATGAAAATCTTAATCATTGAAGACGATT
hp1021^{C}-R	tatggcggaTTATTTGCGCGGTAAGTTATATTTC

hp1021^{C}-CAT-F	cgcgcaaataaTCCGCCATATTGTGTTGAAACA
hp1021^{C}-CAT-R	tagcttcttgGGGCACCAATAACTGCCTTAAAG
hp1021^{C}-down-F	attggtgcccCAAGAAGCTATCGTTTGCTAAACAA
hp1021^{C}-down-R	caggaattcgatatcaagcttAAATACGCTAAAAATAAGCAAATGGA
qPCR	
16s rRNA-qPCR-F	CTCATTGCGAAGGCGACCT
16s rRNA-qPCR-R	TCTAATCCTGTTTGCTCCCCA
ureA-qPCR-F	TTTCGTTGTCTGCTTGCCTATC
ureA-qPCR-R	CGGCTCACACTTCCATTTCTTT
ureB-qPCR-F	TAACTTCACCCACACGACCCAT
ureB-qPCR-R	CCGCTTCCACTAACCCCACTAT
hp1021-qPCR-F	CAACCACACGACTTTCCTTTCCAT
hp1021-qPCR-R	GTTATATTTCCGGCGTTTTTCCCA
fliA-qPCR-F	AAAAAACTGAAACAAGCGAAAAA
fliA-qPCR-R	CACGGCTGGTAAATACTGAATAG
arsR-qPCR-F	ATTATGGGGCTGATGATTACCTC
arsR-qPCR-R	GGCTCACTCACTTCTTCTTTTTT
hp0222-qPCR-F	ATGGAAAAGACAGAAAACACAGA
hp0222-qPCR-R	AAATACAAGCTTACAGCACGAGT
flgR-qPCR-F	AGCCATTGATTCCATTCGTTTAG
flgR-qPCR-R	GCTGTGTTTGTGTGGTTTTTTTA
hup-qPCR-F	AAGCGTGGAGTTGATCGGTTTTG
hup-qPCR-R	CACCCGTTTGTCTTCAGTTTTAT
hspR-qPCR-F	GATTATGATGAACCGCTTTATTT
hspR-qPCR-R	TCGTTTTGATTTTGTCCATGTCT
hsrA-qPCR-F	GGATAACCCTACAAGCGAGGAAG
hsrA-qPCR-R	TCAATCACATTAGAACCCCAAAA
cheY-qPCR-F	GGTAAGGCTGAGGTCATTACGGC

cheY-qPCR-R	TTTTTCTTTCAAAACTTGGGGGG
nikR-qPCR-F	AATCATCCGCTTTTCGGTTTCTT
nikR-qPCR-R	TCGTCATTAGGGTTGTCTTCTGC
crdR-qPCR-F	TTTAGGAGCGAGCGATTATTTGA
crdR-qPCR-R	GATCTCTTTTTTGCCCCTGACAC
rpoD-qPCR-F	AGCGATTGCGTTTCTTATGAGTC
rpoD-qPCR-R	TTTTGCTTTTCTTCGGTTTTTTT
rpoN-qPCR-F	AAGCTCAAATCATTCCCCCTCTCT
rpoN-qPCR-R	AAACTTCGCTCTCTACCCCTAAAA
hrcA-qPCR-F	GTATTCCAACACACAAATCACACG
hrcA-qPCR-R	GGCTCTATAAAATGCAAACCCTTA
hp0564-qPCR-F	AAACGCAATTTTTCAGTGACTTTT
hp0564-qPCR-R	GAATCTTGGTTAGTGCCTTTTTTG
fur-qPCR-F	GAACACCATGATCACATCATTTGT
fur-qPCR-R	TCACTCTCTTGGCATTCTTTACAC
hp0631-qPCR-F	ACTCCGCTTCAATTCACACACCT
hp0631-qPCR-R	TATCCGCTACTTTATCAGCCCCT
hp0632-qPCR-F	ATTAAAAACGGCGTGGTGGAAAA
hp0632-qPCR-R	GCATGGGTCAAAAGAATGGATAG
hp0635-qPCR-F	AACCCCCAATTCTATCGTTTTGT
hp0635-qPCR-R	TGTAGCATTCTGGAGGCTTTTTC
hp0389-qPCR-F	AAAAATTGAAATCATTCAAACGA
hp0389-qPCR-R	CACACATCCACCACTAAAAGCGG
flgL-qPCR-F	TTGGATAATAATGGGGCTTTTTT
flgL-qPCR-R	TTGTTGGTAGATGGCTGGGTCAG
fliS-qPCR-F	GAAAAAGGGGGGGAAGTGGCGGT
fliS-qPCR-R	AGGCGAGTTCATCTGAATGGATT
hp1120-qPCR-F	GCGAGAGTCCTTGTTTGTTTTGA
hp1120-qPCR-R	GGGAATGATTTGTTGGATGAGCG

hp0755-qPCR-F	ACGCTTGAACCCTAAACACAT
hp0755-qPCR-R	CGTAACCGCATTAAAACTCCC
hp1000-qPCR-F	AGCCAATGAAAAAGGAGGGAG
hp1000-qPCR-R	CTGTGCGATTAAAGAGCGTGA
pdxA-qPCR-F	TATTGATGCCCCCTTACCCTT
pdxA-qPCR-R	TCCACCTCTTTACTATCCGCT
hp1582-qPCR-F	TAAGCCCCTTGCCTATCAACA
hp1582-qPCR-R	ACAATCCAATCCCCCCTCTGT
pgi-qPCR-F	ACCTTAACCAACGCCCTAACC
pgi-qPCR-R	CCCCCCTACAAAATCCCAAAA
hp0939-qPCR-F	CGCTCTTGGGGTCTATATGGA
hp0939-qPCR-R	AAAGGCTGATGAAAGTGTTAC
hp1229-qPCR-F	GATCGGCAGAGACGGCAAAAC
hp1229-qPCR-R	GATACTCGCCACCCCAGAATG
hp1036-qPCR-F	CTACGATCATCCTTAAAACATC
hp1036-qPCR-R	TAACACCGAATCCCTTTCACTC
hp0806-qPCR-F	AGTTTTGGGGAGTTGCTCTTAT
hp0806-qPCR-R	GAATGGTTTTTGTGGATCGTGT
trpC-qPCR-F	GGATTTTGTGGGCGTGTTTGTG
trpC-qPCR-R	ATTGAGCGATTTCTTTTTGCGA
hp1589-qPCR-F	TTTTTTTAGAGATGAAGGGGTC
hp1589-qPCR-R	TCAATCAAAGTTGTGTCAGACT
hp1283-qPCR-F	TATGGCGTGTTGAATCTCGGAG
hp1283-qPCR-R	AAAGAAATGGCATTTTTGTGGC
hp0880-qPCR-F	ATAGTCTCATATCAGCTTGGCG
hp0880-qPCR-R	AAATCTTTTTGAATTTTTTCCA
hp0483-qPCR-F	TTCCCTAAACCCATAAAAACGC
hp0483-qPCR-R	CGCACAAATTCCAAAAAAACAT
infB-qPCR-F	GAAGGGAGGGATAGAGATTGTC

infB-qPCR-R	CTTTTTTGGATTTCTTGGAGTT
hp0415-qPCR-F	TTATTATTGTGGTGGGTATTGG
hp0415-qPCR-R	AGGGTTTTTTTTGTAAGTTTCA
hp1010-qPCR-F	TGAAATGAATCACCAACAAGAA
hp1010-qPCR-R	TCCCCACGATAGAATACACCCT
hp0454-qPCR-F	TCATTATCAATCGCGCCATTCC
hp0454-qPCR-R	CCCCAAGCTTTTTGCCAACTCT
hp0264-qPCR-F	ATTCCCAAGGCATTCTCATTCAA
hp0264-qPCR-R	CCATATTCGCCAAAAGATACACA
hp0250-qPCR-F	CAGCCTGAACCCCTTGCATAAAA
hp0250-qPCR-R	GCCCATAGCGATACACACCCTTT
hp0291-qPCR-F	CTCATCAAACAAGAAAGCCCCAT
hp0291-qPCR-R	AACCTCTGTATAAAAACCCGTAA
flhB-qPCR-F	GGCTCATTTCTTCGCTTTTATTT
flhB-qPCR-R	CTTGCTGTTTGTATTCGTCCTTA
hp1231-qPCR-F	AAAGGTGATCAAAACTTTCAACA
hp1231-qPCR-R	CTATACCATAACTAGGAGCGGCG
hp0614-qPCR-F	AAAAACAAGCAAAGAGCGAAGAA
hp0614-qPCR-R	AAAGAGGAGCGAGCGGTTAGAAA
flhA-qPCR-F	GAGGGTTTTTAGTGGGCGTGTT
flhA-qPCR-R	TCTTCTTCATTTTGCGTGGTGC
hp0792-qPCR-F	CTATCCAAGAAGCCAAACAGCG
hp0792-qPCR-R	TAACTCCCCAAAAGCAAACCAC
thiE-qPCR-F	TTCCCACACCATCTAAAAAAGA
thiE-qPCR-R	TAATGACTGCGATACCCCCATA
hemE-qPCR-F	CGTTAATCGGTTTTTGCGGATC
hemE-qPCR-R	CGCTATAAAGCATTTTCTTGCT
hp0653-qPCR-F	TCATGCGACTTTCAATTTCTTG
hp0653-qPCR-R	GATTTCCTGCTTTTAGCGATCC

frdB-qPCR-F	CGGGTGTTGTATCGCTTCTTGC
frdB-qPCR-R	GTTTCTTTCATCGTGGCTGTCA
trmE-qPCR-F	AGACATTCCTAGCGATTTTTTA
trmE-qPCR-R	CTTTATTCCTTTGTTTTTGAGC
atpC-qPCR-F	TAGTGTGGTAGTTCCTGAGGGG
atpC-qPCR-R	AATTGATCGCAATGTGTTCTTT
hp1461-qPCR-F	ATCCATTTTATTGGGCGTTTGC
hp1461-qPCR-R	CGGAGGTCATGATTTCGCTGTT
hp0133-qPCR-F	GTGAAAGCAAGGGAACAAAATA
hp0133-qPCR-R	ATGCCCAAAAAAAGATGAAAAA
hp0389-qPCR-F	CCAAAGCGACTGCCCTAAGCGA
hp0389-qPCR-R	ATTCCAACCAGAGCCAAACAAA
hp0096-qPCR-F	ACAGAATCCGTAGCCCAGCACA
hp0096-qPCR-R	TCCCTAAACCAATAACCCCCCA
hp0013-qPCR-F	CAAGGAATGAAGAAGAAAACCA
hp0013-qPCR-R	TTATCCACCAAACTCAAAGGGC
hp1132-qPCR-F	CCTCAAATGATCGGTGAGAAAC
hp1132-qPCR-R	AAAGAACGGCTGGGATAAAAAC
hp1295-qPCR-F	CGACCTTTAACAACACCAACAT
hp1295-qPCR-R	CTTAGAGCGCTTTCTACAGCCT
hp0633-qPCR-F	ATGTCATGTTTGGGTTTTTGCT
hp0633-qPCR-R	TATACGCTCCTTTAGTGTGGGG
hp1281-qPCR-F	ATGAATGAAGAATCAAAAACGC
hp1281-qPCR-R	CTAAACAAATCCCTAAAATGGG
porA-qPCR-F	CAGCCTATCCTATCACCCCATC
porA-qPCR-R	AAACCTCTACCATTAACGCCAA
hp1380-qPCR-F	ATTGGCTTTGACTTTGGGGCTT
hp1380-qPCR-R	AGGGATATTGCGAATGATTTGC
fbcH-qPCR-F	GGCGTGATTTATTATGGGGTTG

fbcH-qPCR-R	ATAGGGGCTGGAATGTTTTGGG
hp1413-qPCR-F	GACCCCTGAACTAAACCTCAAA
hp1413-qPCR-R	GCGGATAAAAATCACGCCAAAA
hp0510-qPCR-F	ATAGGGTTTTATTTAGAGGGCG
hp0510-qPCR-R	TGATTTCATATTTTTTAGCGGC
hp0147-qPCR-F	GATTGATGGGATAGGGGAGTTT
hp0147-qPCR-R	CATTGTATTGCCCGATTTGAGA
flaG-qPCR-F	TTATTGAGCGAGCGTCTGAATG
flaG-qPCR-R	CACGGCTTCTTTAGAGGGTATTTC
flgK-qPCR-F	GGCTCGTTTGACTTAATCGCTTAC
flgK-qPCR-R	GGCTTGGATAATATCGTTCATGGT
fliD-qPCR-F	CTATTTTGGCTCACACCTCCAATC
fliD-qPCR-R	AAACTCACTTCACTTTTTCCGCTC
motA-qPCR-F	TCAGTCATCATCATCGTGCCTA
motA-qPCR-R	CAACACCCCATCTTTTCTAGCC
motB-qPCR-F	TATCGCTCTTTATGCCATTTCAG
motB-qPCR-R	CTCGCCATTTGTTCTTCTTCTTT
hp1082-qPCR-F	TGAATCTGATCTTACGCCTTTATG
hp1082-qPCR-R	TTGAGTGATGCTCTCTATTTTTTG
hp0607-qPCR-F	CTCTGCTTTTTCTGGGACTTCTC
hp0607-qPCR-R	ACGGGCTTTTAGTTTCTGTGATT
sodB-qPCR-F	GTTGCTCGTTTGAATGATTTCA
sodB-qPCR-R	GACCACTTTGTTTGGCTCTGGT
Expression of Strep-HP1021	
hp1021^{CDS}-F	cccgaattcgagctcggtaccATGAAAATCTTAATCATTGAAGACGATT
hp1021^{CDS}-R	gagaccatggtccccctgcagTTATTTGCGCGGTAAGTTATATTTC
Electrophoretic mobility shift assay	

P*ureA*-F	CCATTATCACTCCAATTTTAATTCTCA
P*ureA*-R	CTTATTCTCCTATTCTTAAAGTGTTTTTCC
P*ureA*-AF700-R	CTTATTCTCCTATTCTTAAAGTGTTTTTCC
DNase I footprinting assay	
pLB-P*ureA*-F	AAGAACATCGCTTTTCGATGGCAG
pLB-P*ureA*-FAM-R	CGACTCACTATAGGGAGAGCGTC
Site-directed mutagenesis	
pLB-P*ureA*mt-F	gcttcgtcagtcgaaacatgtacgtgATTACATCCAACCTTGATTTCGTTAT
pLB-P*ureA*mt-R	tgtttcgactgacgaagccggctgGAAGCGTTCATTAACTAATTATTATTTAAAA

The lowercase letters represent overlapping nucleotides.
