# Supplementary material for: AI-2 Induces Urease Expression Through Downregulation of Orphan Response Regulator HP1021 in Helicobacter pylori
Source: Front Med (Lausanne). 2022 Apr 1;9:790994. doi: 10.3389/fmed.2022.790994 (PMC9010608; doi:10.3389/fmed.2022.790994)
Supplement: Supplementary file 3 [file Table_2.pdf]

Supplementary Table 2. Validation of gene expression by qPCR (Wild type/Δ*luxS*).

Function	Gene name	log_2FoldChange (RNAseq)	log_2FoldChange (qPCR)	Significance
Aromatic compound biosynthetic	HP1120	2.90	1.89	TRUE
	RpoN (HP0714)	1.87	1.22	TRUE
	HP0755	1.76	0.76	TRUE
	PdxA (HP1583)	1.57	0.64	TRUE
	HP1582	1.55	0.64	TRUE
	Pgi (HP1166)	1.33	1.56	TRUE
	HP1229	1.25	0.79	TRUE
	HP1036	1.24	0.84	TRUE
	HP0806	1.19	0.58	TRUE
	TrpC (HP1279)	1.15	0.10	FALSE
	HP1589	1.10	0.42	TRUE
	HP0483	1.08	0.81	TRUE
	HP0415	1.03	0.58	TRUE
	HP0454	1.01	0.43	FALSE
	HP0264	1.01	1.09	TRUE
	HP0250	0.99	0.12	FALSE
	HP0291	0.96	0.19	FALSE
	HP1231	0.88	0.64	TRUE
	HP0614	0.84	0.36	FALSE
	HP0792	0.80	-0.01	FALSE
	HrcA (HP0111)	0.79	1.03	TRUE
	ThiE (HP0843)	0.76	0.71	TRUE
	HemE (HP0604)	0.72	0.17	FALSE
Peptide transport	FlgK (HP1119)	2.77	1.22	TRUE
	HP1000	1.58	0.74	TRUE
	HP0939	1.33	0.97	TRUE
	HP1283	1.09	0.40	TRUE
	HP0880	1.09	0.49	TRUE
	HP1010	1.02	0.29	TRUE
	HP0454	1.01	0.43	FALSE
	HP0264	1.01	1.09	TRUE
	HP0250	0.99	0.12	FALSE
	FlhB (HP0770)	0.92	0.69	TRUE
	FlhA (HP1041)	0.82	0.54	FALSE
Amine metabolic process	UreB (HP0072)	1.37	0.74	TRUE
	TrpC (HP1279)	1.15	0.10	FALSE
	InfB (HP1048)	1.07	1.18	TRUE
Oxidoreductase activity	HP0653	-2.07	-0.73	TRUE
	FrdB (HP0191)	-1.39	-0.80	TRUE

Function	Gene name	log_2FoldChange (RNAseq)	log_2FoldChange (qPCR)	Significance
Oxidoreductase activity	HP1461	-1.29	-0.81	TRUE
	HP0133	-1.02	-0.90	TRUE
	HP0389	-1.10	-0.79	TRUE
	HP0096	-1.04	-1.16	TRUE
	HP1295	-0.95	-0.79	TRUE
	HP0633	-0.94	-0.51	TRUE
	PorA (HP1110)	-0.82	-0.56	TRUE
	HP1380	-0.80	-1.16	TRUE
	FbcH (HP1538)	-0.74	-0.43	TRUE
	HP1413	-0.73	-0.81	TRUE
	HP0510	-0.72	-0.44	TRUE
	HP0147	-0.71	0.01	FALSE
Drug metabolic process	HP0653	-2.07	-0.73	TRUE
	TrmE (HP1452)	-1.37	-0.62	TRUE
	AtpC (HP1131)	-1.30	-1.46	TRUE
	HP1132	-0.96	-1.39	TRUE
	HP1281	-0.91	-1.19	TRUE
Alpha-amino acid metabolic process	HP0653	-2.07	-0.73	TRUE
	HP0013	-1.02	-0.90	TRUE
	HP1281	-0.91	-1.19	TRUE
	HP1380	-0.80	-1.16	TRUE
	HP0510	-0.72	-0.44	TRUE
ATP metabolic process	TrmE (HP1452)	-1.37	-0.62	TRUE
	AtpC (HP1131)	-1.30	-1.46	TRUE
	HP1132	-0.96	-1.39	TRUE
Flagellar related genes	FlgL (HP0295)	1.07	0.74	TRUE
	FliS (HP0753)	1.30	1.18	TRUE
	FlaG (HP0751)	2.37	1.15	TRUE
	FlgK (HP1119)	2.77	1.22	TRUE
	FliD (HP0752)	2.35	0.97	TRUE
	MotA (HP0815)	1.22	0.74	TRUE
	MotB (HP0816)	1.35	0.76	TRUE
Iron related cellular activity	HP0631	-1.16	-1.27	TRUE
	HP0632	-1.04	-1.55	TRUE
	HP0635	-1.04	-0.74	TRUE
	SodB (HP0389)	-1.10	-0.91	TRUE
Multidrug efflux pump	HP1082	1.54	0.89	TRUE
	HP0607	1.31	1.40	TRUE

Student's *t*-test was used to evaluate the statistical significance. True represents statistically significant with p value < 0.05, False represents statistically non-significant.
