# Supplementary material for: AI-2 Induces Urease Expression Through Downregulation of Orphan Response Regulator HP1021 in Helicobacter pylori
Source: Front Med (Lausanne). 2022 Apr 1;9:790994. doi: 10.3389/fmed.2022.790994 (PMC9010608; doi:10.3389/fmed.2022.790994)
Supplement: Supplementary file 4 [file Table_3.pdf]

Supplementary Table 3. Differential expression of transcriptional regulators identified by RNAseq.

Gene ID	Log_2FoldChange (Δ*luxS*/Wild type)	Significance
HP1021	-1.4661	TRUE
Fur(HP1027)	-0.8380	TRUE
HP0564	-0.8293	TRUE
CheY(HP1067)	-0.3501	FALSE
HP0222	-0.2007	FALSE
FlgR(HP0703)	-0.1540	FALSE
FliA(HP1032)	-0.1357	FALSE
CrdR(HP1365)	-0.0988	FALSE
RpoD(HP0088)	-0.0968	FALSE
NikR(HP1338)	-0.0105	FALSE
ArsR(HP0166)	0.1556	FALSE
Hup(HP0835)	0.1783	FALSE
HsrA(HP1043)	0.2946	FALSE
HspR(HP1025)	0.6418	FALSE
HrcA(HP0111)	0.7870	TRUE
RpoN(HP0714)	1.8690	TRUE

True represents statistically significant with p value < 0.05, false represents statistically non-significant.
